# The inhibition of enterocyte proliferation by lithocholic acid exacerbates necrotizing enterocolitis through downregulating the Wnt/β‐catenin signalling pathway

**DOI:** 10.1111/cpr.13228

**Published:** 2022-04-20

**Authors:** Zhoushan Feng, Chunhong Jia, Xiaojun Lin, Hu Hao, Sitao Li, Fei Li, Qiliang Cui, Yaoyong Chen, Fan Wu, Xin Xiao

**Affiliations:** ^1^ Department of Pediatrics Sun Yat‐sen University Sixth Affiliated Hospital Guangzhou China; ^2^ Department of Pediatrics The Third Affiliated Hospital of Guangzhou Medical University Guangzhou China; ^3^ Key Laboratory for Major Obstetric Diseases of Guangdong Province Guangzhou China

## Abstract

**Objectives:**

Necrotizing enterocolitis (NEC) is a catastrophic gastrointestinal emergency in preterm infants, whose exact aetiology remains unknown. The role of lithocholic acid (LCA), a key component of secondary bile acids (BAs), in NEC is unclear.

**Methods:**

Clinical data were collected to analyse the changes of BAs in NEC patients. In vitro studies, the cell proliferation and cell death were assessed. In vivo experiments, the newborn rats were administered with low or high dose of LCA and further induced NEC.

**Results:**

Clinically, compared with control group, total BAs in the NEC patients were significantly higher when NEC occurred. In vitro, LCA treatment significantly inhibited the cell proliferation through arresting cell cycle at G1/S phase without inducing apoptosis or necroptosis. Mechanistically, the Wnt/β‐catenin pathway was involved. In vivo, LCA inhibited intestinal cell proliferation leading to disruption of intestinal barrier, and thereby increased the severity of NEC. Specifically, LCA supplementation caused higher levels of FITC‐labelled dextran in serum, reduced PCNA expression and inhibited the activity of Wnt/β‐catenin pathway in enterocytes. The LC–MS/MS test found that LCA was significantly higher in intestinal tissue of NEC group, and more obviously in the NEC‐L and NEC‐H group compared with the DM group.

**Conclusion:**

LCA exacerbates NEC by inhibiting intestinal cell proliferation through downregulating the Wnt/β‐catenin pathway.

## Graphical abstract

The role of lithocholic acid (LCA), a key component of bile acids, in necrotizing enterocolitis(NEC) remains unclear. Our data indicate that LCA exacerbates NEC by inhibiting enterocyte proliferation through downregulating the activity of Wnt/β‐catenin pathway.

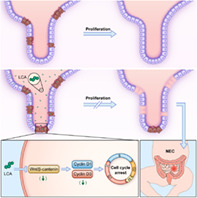

## 1 INTRODUCTION

Necrotizing enterocolitis (NEC) is a catastrophic disease that predominantly occurs in preterm infants. Approximately 7% of infants with a birth weight between 500 and 1500 g develop NEC, with an overall mortality rate of 10%–50%.^1^, ^2^ According to our previous retrospective analysis from 2008 to 2017 in Guangdong province, the incidence of NEC in very preterm infants was 10.1%.^3^ This disease can be mild to severe with clinical symptoms including abdominal distension, pneumatosis intestinalis, bowel perforation, sepsis, shock and even death.^4^ Survivors of severe episodes of NEC frequently suffer serious sequelae such as short bowel syndrome, cholestasis and neurodevelopmental retardation.^5^, ^6^ Although prematurity,^7^ enteral feeding,^8^, ^9^ intestinal bacterial colonization,^10^, ^11^ and immune imbalance,^12^ are considered as major risk factors, the pathophysiology of NEC is still poorly understood. The intestinal epithelial cells (IECs), immune system and microbiome, coordinate with each other to maintain normal intestinal homeostasis and barrier integrity.^13^ In response to microbial invasion, the death of IECs increases, but more importantly, there is an enhanced proliferation and renewal to retain the intestinal barrier and tissue homeostasis. However, an impairment of IEC proliferation leads to increased intestinal permeability and barrier dysfunction.

The Wnt/β‐catenin pathway, a multitasking and evolutionary conserved pathway, plays essential roles in embryonic development, tissue homeostasis and regeneration.^14^, ^15^ β‐Catenin is a key component of the Wnt signalling cascade; the levels and activity of this protein are tightly regulated by a destruction complex consisting of the enzyme glycogen synthase kinase‐3β (GSK‐3β), the adenomatosis polyposis coli (APC) protein and the scaffolding protein AXIN.^16^ β‐Catenin can be phosphorylated by the destruction complex through GSK‐3β and degraded by the ubiquitination‐proteasome pathway in the cytoplasm, thereby failing to enter the nucleus to promote the transcription of target genes, including cyclin D1, c‐jun and c‐myc.^17^ These target genes are well known to play important roles in the proliferation of intestinal cells.^18^, ^19^, ^20^ In addition, it has been reported that impairment of the Wnt/β‐catenin pathway can lead to the dysfunction of intestinal regeneration during NEC.^21^ However, the regulatory factors of the Wnt/β‐catenin pathway in the process of NEC remain unclear.

Bile acids (BAs) are important in absorption of dietary fats in intestine. The primary BAs, cholic acid (CA) and chenodeoxycholic acid (CDCA), are mainly derived from the metabolism of cholesterol by hepatocyte, which are secreted in the bile conjugated to taurine or glycine. Most of the BAs are reabsorbed in the ileum and enter the enterohepatic circulation. BAs that escape reabsorption are metabolized by the intestinal microflora to produce secondary BAs, deoxycholic acid (DCA) and lithocholic acid (LCA), from CA and CDCA, respectively. DCA accumulates in large amounts in the enterohepatic circulation pool, but only a small amount of LCA is absorbed in the ileum and excreted into bile. It has been confirmed that changes in the composition of the BA pool are the hallmarks of many gastrointestinal diseases.^22^, ^23^ The most vulnerable segment of the intestine during NEC is the distal ileum and proximal colon, where BAs are reabsorbed. The accumulation of secondary BAs in the intestine can cause damage to the intestinal epithelium.^24^, ^25^, ^26^ It has been reported that DCA is closely related to NEC by regulating cell proliferation or the BA transporter.^27^, ^28^ Similarly, LCA has been found to be elevated in intestinal bile salts from patients with NEC.^29^ However, the role and mechanisms of LCA in NEC remains clear. In this study, we aimed to unravel the impact of LCA in the pathogenesis of NEC.

## 2 MATERIALS AND METHODS

### 2.1 Clinical data collection

Clinical data were obtained between April 2021 and October 2021 in accordance with the clinical research and applied ethics committee of the Third Affiliated Hospital of Guangzhou Medical University. Written informed consent was obtained from the patient's parents. The following data were collected, including gestational age (GA), birth weight (BW), gender, Apgar scores at 5 min, total BAs within 24 h after birth, as well as total BAs, total bilirubin and direct bilirubin at the time of NEC occurrence. In addition, treatment with red blood cell (RBC) or nonsteroidal anti‐inflammatory drugs (NASIDs), including ibuprofen or indomethacin, was also collected, which are risk or protective factors for NEC.^30^, ^31^ The diagnosis of NEC was defined as Bell's stage ≥II with radiographic evidence of NEC.^32^ In this study, infants with NEC were matched by GA, BW and days of age at collection of total BAs, with a maximum allowable deviation of 10% to two controls.^33^

### 2.2 Cell culture

Rat intestinal epithelial IEC‐6 cells were obtained from the American Type Culture Collection (ATCC; Rockville, MD). Cells were cultured in Dulbecco's Modification of Eagle's Medium (DMEM; Corning, NY) supplemented with 10% fetal bovine serum (Gibco, Grand Island, NY). Cells were maintained at 37°C in a humidified atmosphere containing 5% CO_2_.

### 2.3 Cell proliferation assay

Cells (3000 cells/well) were seeded in 96‐well plates overnight and treated with different doses of CA, DCA and LCA (Sigma‐Aldrich, St. Louis, MO) and with or without caspase inhibitors (Z‐VAD‐FMK and Z‐DEVD‐FMK), necroptosis inhibitors (necrostatin‐1) (Selleck, Shanghai, China) or β‐catenin agonist (Wnt‐3A) (R&D Systems, Minneapolis, MN). Cell proliferation was determined by cell counting kit‐8 (CCK‐8) assays (Dojindo, Kumamoto, Japan) in accordance with the manufacturer's instructions. The optical density (OD) at 450 nm was tested using BioTek ELx800 microplate reader (BioTek, Winooski, VT). All assays were performed in triplicate.

### 2.4 RNA sequencing

RNA sequencing was performed by Novogene (Suzhou, China). In brief, RNA quantification and identification were determined by an RNA Nano 6000 assay kit and a Bioanalyzer 2100 system (Agilent Technologies, CA). Then, cDNA library construction and quality assessment of the library were performed with an AMPure XP system (Beckman Coulter, Beverly, MA). Qualified libraries were sequenced on an Illumina Novaseq platform and 150 bp paired‐end reads were generated. Differentially expressed genes (DEGs) were identified using the edger R package with a log2 |fold change| >1 and adjust *p*‐value (*p*adj) ≤0.05. KEGG pathway enrichment analysis of DEGs was performed using the clusterprofiler R package. Gene set enrichment analysis (GSEA), a computational method used to determine whether a pre‐defined gene set can show significant concordant differences between two biological genes, was also carried out.

### 2.5 Cell apoptosis assays

The annexin V‐FITC/propidium iodide (PI) cell apoptosis kit (Tianjin Sungene Biotech, Tianjin, China) was used to quantify cell apoptosis and necroptosis in accordance with the manufacturer's instructions. In brief, IEC‐6 cells were collected and washed twice with phosphate buffered saline (PBS) (pH 7.4), followed by resuspension in staining buffer. Thereafter, 5 μl of PI and 5 μl of Annexin V‐FITC were mixed with the cells. After incubation at room temperature for 5 min, the cells was analysed using an Attune Nxt Acoustic Focusing Flow Cytometer (Invitrogen, Thermo Fisher Scientific, MA).

### 2.6 Cell cycle assays

After treatment with different doses of LCA for 24 h, the cells were harvested and fixed with ethanol (70%) overnight at 4°C. Next, the cells were washed once with PBS and then incubated in 500 μl of PI/RNase (ratio 9:1; KeyGEN BioTECH, Nanjing, China) working solution for 1 h in the dark. Afterwards, the cell cycle was detected by an Attune Nxt Acoustic Focusing Flow Cytometer (Invitrogen, Thermo Fisher Scientific).

### 2.7 Immunofluorescence

IEC‐6 cells were seeded on coverslips, fixed in 4% paraformaldehyde for 15 min and permeabilized in 0.15% Triton‐100X for 10 min. Then, cells were blocked with 5% goat serum for 1 hour. Anti‐Ki67 or anti‐β‐catenin (CST, Danvers) antibody was then added at a dilution of 1:100 in 1% BSA and incubated overnight at 4°C. Then, cells were washed three times with PBS and incubated with FITC‐labelled secondary antibodies at a dilution of 1:100 in 1% BSA for 2 h in the dark. Finally, 4′,6‐diamidino‐2‐phenylindole (DAPI; Invitrogen, Camarillo, CA) was used for DNA staining. The stained cells were then observed with a Nikon Ni‐U microscope (Nikon, Tokyo, Japan).

### 2.8 Western blotting

Preparation of whole cell lysates and western blotting analysis was performed as previously described.^34^, ^35^ Primary antibodies against cyclin A2 (1:1000), cyclin B1 (1:1000), cyclin D1 (1:1500), cyclin D2 (1:1500), cyclin D3 (1:1500), cyclin E1 (1:1000), CDK2 (1:1500), CDK4 (1:1500), CDK6 (1:1500), CDC2 (1:1500), β‐catenin (1:1500), GSK‐3β (1:2000), p‐GSK‐3β (Ser 9) (1:2000), p‐AKT (Ser 473) (1:2000), c‐jun (1:1500), c‐myc (1:1500), PCNA (1:1500), PARP (1:1500), cleaved‐PARP (1:1500), pro‐caspase 3 (1:1500), cleaved‐caspase 3 (1:1500), RIPK 1 (1:1500), p‐RIPK 1 (Ser166) (1:1500), ERK 1/2 (1:2000), p‐ERK (Thr202/Tyr204) (1:2000), p‐SAPK/JNK (Thr183/Tyr185) (1:1500) and p‐p38 MAPK (Thr180/Tyr182) (1:2000) were purchased from Cell Signalling Technology (Danvers, MA). An antibody against AKT (1:500) was obtained from Santa Cruz Biotechnology (Santa Cruz, CA) and antibodies against β‐actin (1:2000) and β‐tubulin (1:2000) were acquired from Beijing Ray Antibody Biotech (Beijing, China). The grey values of proteins expression in Western blotting were quantified using the Image J software (NIH Image, Bethesda, MD).

### 2.9 Generation of a rat model of intestinal injury

Pregnant rats were obtained from Guangdong Medical Laboratory Animal Center (Guangzhou, Guangdong, China). All animal procedures were approved by the Animal Care and Use Committee of the of Guangzhou Medical University. Newborn rats were randomly divided into three groups immediately after birth: DM, CON‐L and CON‐H groups (seven rats in each group). The DM group was left with their mothers and fed breast milk as normal control. The CON‐L and CON‐H groups were fed with cow's milk‐based rat milk substitute formula (15 g of PreNAN LBW infant formula (Nestle, Netherlands) in 75 ml Esbilac puppy milk replacer (Pet‐Ag Inc., Hampshire, IL)) with LCA at final concentrations of 10 or 20 mM, respectively.

### 2.10 Experimental NEC induction and evaluation

Newborn rats were randomly divided into four groups immediately after birth: DM, NEC, NEC‐L and NEC‐H (seven rats in each group). The DM group was left with their mothers and fed breast milk as normal control. The NEC, NEC‐L and NEC‐H groups were fed with cow's milk‐based rat milk substitute formula and were subjected to asphyxia (breathing 100% nitrogen for 60 s) followed by cold stress (4°C for 10 min) twice a day for 4 days to induce experimental NEC.^36^, ^37^, ^38^ The substitute formula for the NEC‐L and NEC‐H groups were mixed with LCA at final concentrations of 10 and 20 mM, respectively. After 4 days, all surviving rats were euthanized. The intestines were carefully removed, formalin‐fixed, paraffin‐embedded, microtome‐sectioned, stained with haematoxylin–eosin (H&E) and histologically evaluated by two blinded pathologists independently based on an established histology damage scoring system.^39^, ^40^, ^41^ Damage scores ≥2 were considered to have developed experimental NEC.

### 2.11 BAs composition assay

In rats, the composition of BAs in terminal ileum tissue were detected using a liquid chromatography tandem mass spectrometry (LC–MS/MS) system (BioNovoGene, Suzhou, China). In brief, approximately 50 mg of lyophilized homogenized tissue was weighed, ground and methanol was added to precipitate protein. After vertexing for 1 min and centrifugation at 4°C, the supernatant was concentrated and dried in a vacuum; the residue was then redissolved with 100 μl methanol. Finally, the supernatant was analysed with a LC–MS/MS system.

### 2.12 Immunohistochemistry

Immunohistochemical analysis was completed as described previously.^42^ In brief, after deparaffinization, antigen retrieval and background blocking, sections were incubated with anti‐PCNA at 4°C overnight. The next morning, the sections were washed with PBS and then incubated with a HRP conjugated secondary antibody at room temperature for 1 h. Immunoreactivity was then observed by staining with 3,3′‐diaminobenzidine (DAB; Vector Laboratories, Burlingame, CA). Finally, the slides were counterstained with H&E. To quantify immunoreactivity, 15 consecutive non‐overlapping fields at ×400 magnification were scored blindly.

### 2.13 Measurement of intestinal barrier permeability

Four thousand dalton fluorescent dextran–FITC (DX‐4000–FITC; Sigma‐Aldrich) was used to measure intestinal barrier permeability as described previously.^43^, ^44^ In brief, DX‐4000‐FITC (80 mg/100 g body weight, 40 mg/ml) was administered after the rats were fasted for 8 h. Four hours later, blood was taken from the heart and the upper plasma was collected by centrifugation. The level of DX‐4000‐FITC in the plasma was then measured using a Synergy H1 microplate reader (BioTek, Winooski, VT) (excitation, 480 nm; emission, 520 nm). The concentration of DX‐4000‐FITC was then calculated based on a standard curve.

### 2.14 Statistical analysis

Statistical analysis was performed using the SPSS 22 software (SPSS, Chicago, IL) and the GraphPad Prism 7 software (La Jolla, CA). Unless otherwise stated, results are expressed as mean ± standard deviation (SD). Statistical differences between two groups were analysed using the Student's *t*‐test or Wilcoxon test, while multiple groups were evaluated using one‐way analysis of variance (ANOVA) or the Kruskal–Wallis test, and rates were compared and analysed using chi square or Fisher's exact tests. *p* <0.05 was considered statistically significant.

## 3 RESULTS

### 3.1 Elevated total BAs were associated with the clinical risk of NEC

In total, 16 NEC infants and 32 matched non‐NEC infants were included in our study. The demographic and clinical characteristics of the patients were shown in Table 1. In the NEC group, 9 infants were diagnosed with stage II NEC, 7 infants were diagnosed with stage III NEC and 7 infants required surgeries. There were no significant differences in GA, BW, gender, Apgar score at 5 min, enteral feeding within 24 h, type of feeding, total bilirubin, direct bilirubin, time at blood test and NASIDs, except for RBC transfusion between NEC and non‐NEC infants. Interestingly, compared with the control group, total BAs in the NEC group were similar at birth but were significantly increasing when NEC occurred. However, no significant differences in total BAs were observed between patients with stage II and stage III NEC or between surgical and non‐surgical patients (Tables S1 and S2). Overall, high BAs were closely related to NEC and likely to increase the risk of the disease.

**TABLE 1:** Clinical characteristics of NEC and non‐NEC patients

Characteristic	non‐NEC (*n* = 32)	NEC (*n* = 16)	*p* value
GA (weeks)	31.81 ± 3.34	31.6 5 ± 3.51	0.408
BW (g)	1519.56 ± 617.6	1490.00 ± 590.2	0.8789
Female (%)	16/32 (50%)	10/16 (62.5%)	0.413
Apgar score at 5 min	9 (10, 10)	9 (10, 10)	0.966
Enteral feeding within 24 h (%)	26/32 (81.25%)	10/16 (62.5%)	0.157
Type of feeding:			
MM	2/32 (6.25%)	0	0.57
PF	10/32 (31.25%)	6/16 (37.5%)	
MM + PF	20/32 (62.5%)	10/16 (62.5%)	
Total BAs within 24 h after birth (μmol/L)	7.64 (6.07, 9.38)	6.28 (5.01, 9.87)	0.962
Total BAs at NEC diagnosis (μmol/L)	7.91 (6.68, 11.97)	12.90 (10.0, 18.71)	0.007
Total bilirubin (μmol/L)	114.14 ± 44.05	104.15 ± 42.16	0.4563
Direct bilirubin (μmol/L)	11.76 ± 5.83	14.36 ± 6.52	0.1831
Time at blood test (*d*)	10 (7, 15.5)	10 (7.75, 14)	0.057
RBC transfusion (%)	4/32 (12.50%)	8/16 (50.00%)	0.013
NASIDs (%)	1/32 (3.13%)	2/16 (12.5%)	0.527
Surgery (%)	0	7/16 (43.75)	‐

*Note*: Data are expressed as medians (25%, 75%), mean ± SD or numbers (%). *p* values were derived from Student's *t*‐test, Wilcoxon test or chi square.

Abbreviations: BAs, bile acids; BW, birth weight; GA, gestational age; MM, mother's milk; NEC, necrotizing enterocolitis; PF, preterm formula; RBC, red blood cell.

### 3.2 LCA significantly inhibited the proliferation of IECs in vitro

**FIGURE 1:**
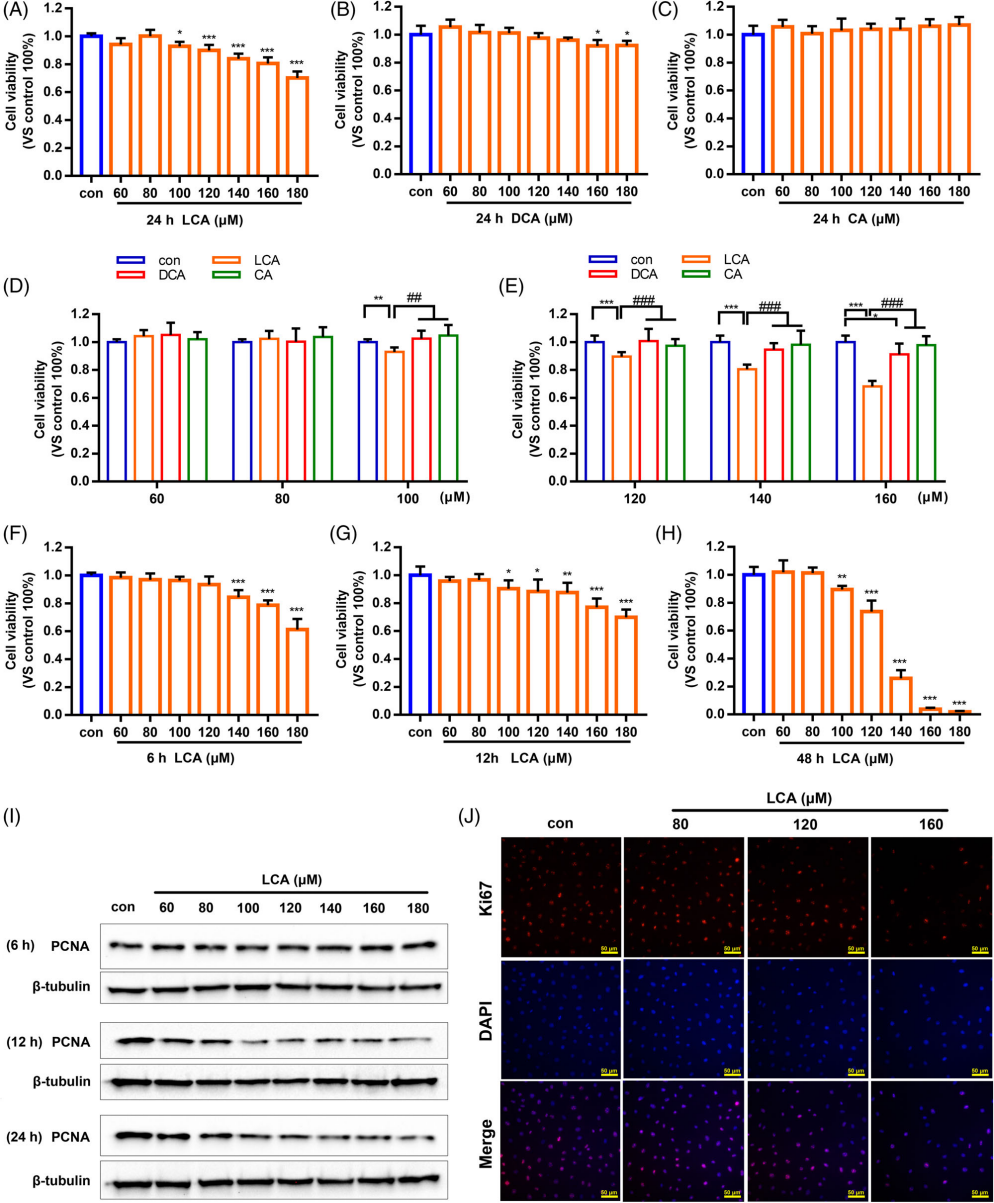
Lithocholic acid (LCA) significantly inhibited intestinal epithelial cell proliferation in vitro. (A–C) IEC‐6 cells were treated with LCA, deoxycholic acid (DCA) or cholic acid (CA) at dose of 60–180 μM for 24 h, respectively, and cell proliferation was tested by CCK‐8 assay. (D,E) IEC‐6 cells were treated with LCA, DCA and CA at same dose of 60–160 μM for 24 h, and cell proliferation were tested by CCK‐8 assay. (F–H) Treatment of IEC‐6 cells with different doses of LCA (60–180 μM) for different times (6, 12 or 48 h), the cell proliferation was examined using CCK‐8 assay. (I) The expression of PCNA in the presence of different dose of LCA (60–180 μM) for 6, 12 or 24 h was determined by western blot. (J) Immunofluorescence staining of Ki67 (Red) after treatment with 80, 120 and 160 μM LCA for 24 h (×400). Cells were counterstained with DAPI (blue). *p <0.05, **p <0.01, ***p <0.001 compared to control by one‐way ANOVA; ^##^ *p* <0.01, ^###^ *p* <0.001 compared to LCA by Student's *t*‐test; ns, not significant. Data provided are the mean ± SD from at least three independent experiments, and bar graphs represent the mean with error bars indicating SD. CA, cholic acid; DCA, deoxycholic acid; LCA, lithocholic acid

As mentioned above, the balance of IEC proliferation and death is critical for preserving intestinal homeostasis. The reduced proliferation of IECs leads to increased intestinal permeability and barrier dysfunction. IEC‐6 cells, small intestine crypt epithelial cells of rats, were treated with different doses (60–180 μM) of CA, DCA or LCA for 24 h, respectively, then CCK‐8 assays to evaluate proliferation. As shown in Figure 1A, compared with the control, LCA significantly inhibited cell proliferation in a dose‐dependent manner. In contrast, DCA only inhibited cell proliferation at high concentrations (160 or 180 μM) (Figure 1B) and CA did not have any effect (Figure 1C). Moreover, the IEC‐6 cells were administered with different doses (60–160 μM) of CA, DCA and LCA for 24 h at the same time. The results suggested that IEC‐6 cell proliferation decreased more significantly in the LCA treatment than DCA or CA treatments (Figure 1D,E). Furthermore, similar inhibitory effects were obtained at other times (6, 12 or 48 h) (Figure 1F–H). PCNA and Ki67 are important indicators of cell proliferation; we found that the expression of PCNA decreased in a dose‐dependent manner under treatment with different does of LCA (60–180 μM) for 12 and 24 h (Figures 1I and S1A–C). In addition, the immunofluorescence staining of Ki67 decreased sharply when IEC‐6 cells were treated with 80, 120 and 160 LCA for 24 h (Figure 1J). Collectively, these results indicated that LCA significantly inhibited the proliferation of IEC‐6 cells in vitro.

### 3.3 The inhibition of cell proliferation was caused by cell cycle arrest at the G1/S phase

**FIGURE 2:**
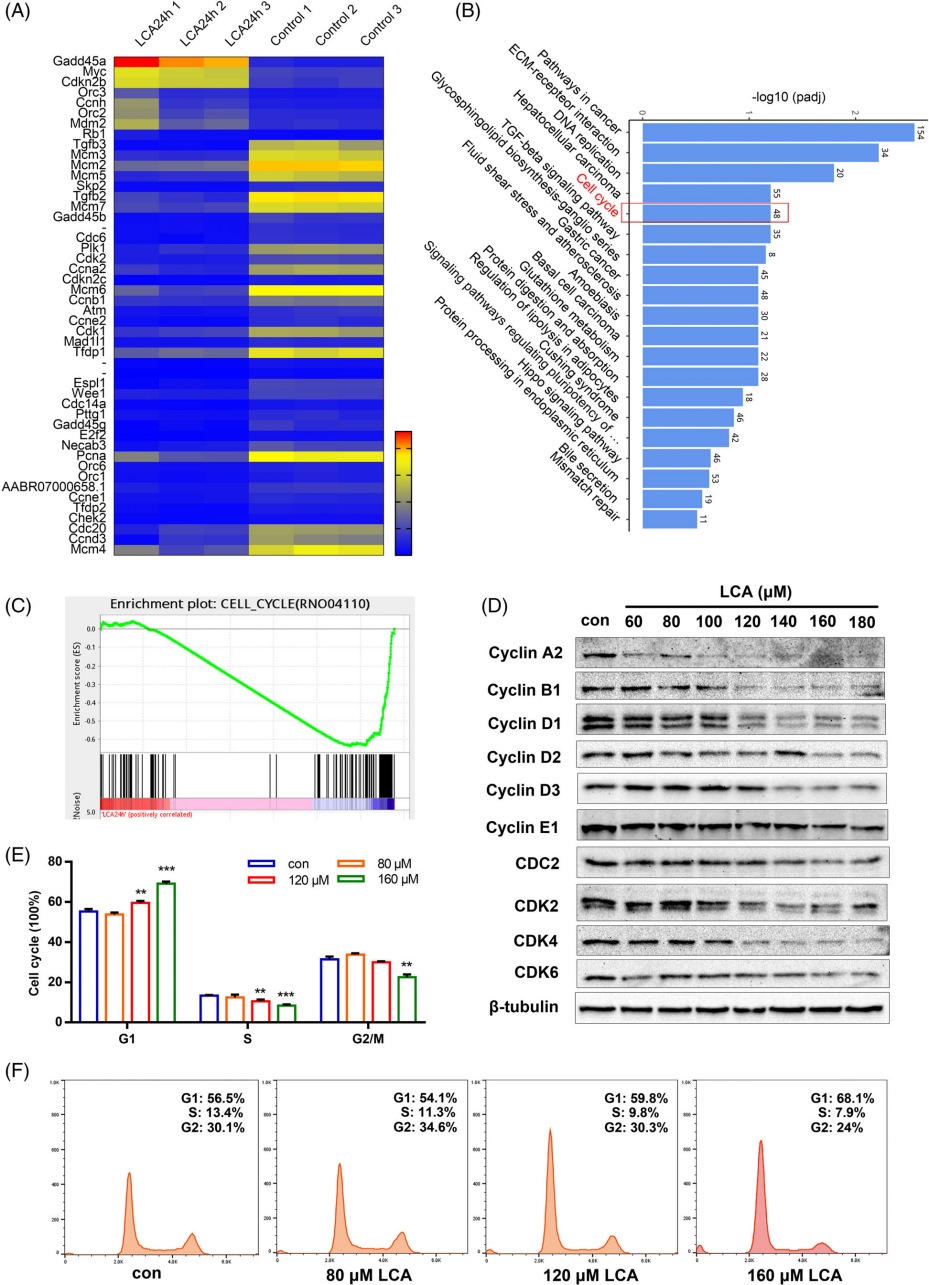
Lithocholic acid (LCA) led to cell cycle arrest at G1/S phase. IEC‐6 cells were treated with 120 μM LCA for 24 h and detected by RNA sequencing. (A) Heat map of DEGs related to cell cycle. (B) Bar graph of the top 20 results from the KEGG enrichment analysis ranked by −log10 (*p*adj). (C) Gene set enrichment analysis revealed negative enrichment of cell cycle. (D) Expression of cyclins and cyclin‐dependent kinases under treatment with different dose of LCA (60–180 μM) for 24 h. (E,F) Cell cycle progression was assessed by flow cytometry after treatment of cells with 80, 120 and 160 μM LCA for 24 h. *p <0.05, **p <0.01, ***p <0.001 compared to control by one‐way ANOVA. Data provided are the mean ± SD from at least three independent experiments, and bar graphs represent the mean with error bars indicating SD

RNA sequencing was used to investigate the underlying mechanisms induced by LCA treatment. A total of 1731 up‐regulated genes and 2198 down‐regulated genes were identified after LCA treatment (Figure S2A). Of these, 48 DEGs related to the cell cycle were identified (Figure 2A). KEGG pathway analysis indicated that LCA was associated with cell cycle (Figures 2B and S2B). GSEA also indicated that genes related to cell cycle progression were significantly repressed by LCA treatment (Figure 2C).

Next, we investigated key regulatory proteins involved with the cell cycle. We found that treatment of IEC‐6 cells with LCA (60–180 μM) for 24 h led to a dose‐dependent reduction in the expression of cyclin D1, cyclin D3, cyclin A2, cyclin B1, cyclin E1, CDC2, CDK2, CDK4 and CDK6 (Figure 2D, Figure S3A–J). Similar results were obtained after both 12 and 6 h of treatment (Figure S2C,D). Furthermore, we used flow cytometry to determine which phase of the cell cycle was arrested. As shown in Figure 2E,F, in the presence of different concentrations of LCA (80, 120 and 160 μM), the cell cycle was arrested at the G1/S phase; the proportion of cells in G1 phase was 54.1%, 59.8% and 68.1%, respectively. Collectively, these data suggested that LCA inhibited IEC‐6 cell proliferation by arresting the cell cycle in G1/S phase.

### 3.4 LCA did not significantly induce apoptosis or necroptosis

**FIGURE 3:**
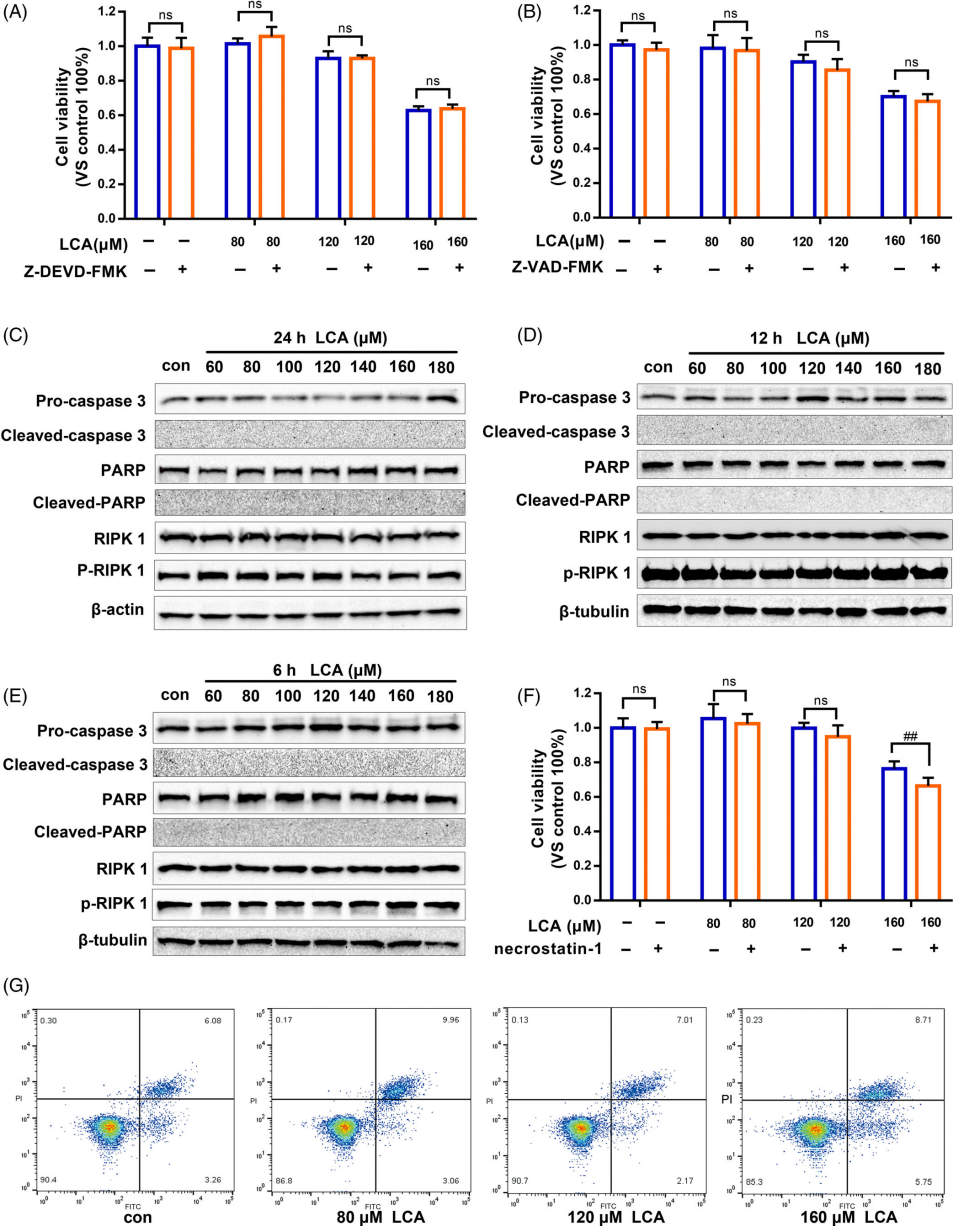
Lithocholic acid (LCA) did not drastically induce apoptosis or necroptosis. (A,B) IEC‐6 cell proliferation was tested by CCK‐8 assay under treatment with Z‐DEVD‐FMK (50 μM) or Z‐VAD‐FMK (50 μM), respectively for 1 h, following by LCA (80, 120 and 160 μM) for 24 h. (C–E) IEC‐6 cells treated with different dose of LCA (60–180 μM) for 24, 12 or 6 h, and the level of pro‐caspase 3, cleaved‐caspase 3, PARP, cleaved‐PARP, RIPK1 and p‐RIPK 1 (Ser166) were detected by western blot. **(**F**)** IEC‐6 cell proliferation was tested by CCK‐8 assay under treatment with necrostatin‐1 (50 μM) for 1 h, following by LCA (80, 120 and 160 μM) for 24 h. **(**G**)** Apoptosis and necroptosis were assessed by annexin V‐FITC and PI double staining using flow cytometry, after cells were treated with 80, 120 and 160 μM LCA for 24 h, respectively. ^##^ *p* <0.01 compared to LCA by Student's *t*‐test; ns: not significant. Data provided are the mean ± SD from at least three independent experiments, and bar graphs represent the mean with error bars indicating SD

Cell proliferation and cell death are essential yet opposing cellular processes and are critical for tissue homeostasis and intestinal function. CCK‐8 assays showed that Z‐DEVD‐FMK and Z‐VAD‐FMK, inhibitors of apoptosis, did not significantly restore the cell viability of IEC‐6 cells treated with different doses of LCA for 24 h (Figure 3A,B). Under treatment with LCA (60–180 μM) for 24, 12 or 6 h, cleaved‐caspase 3 and cleaved‐PARP were not detected by western blotting (Figure 3C–E). Necroptosis is a newly recognized form of programmed necrosis.^45^ Necrostatin‐1, a necroptosis inhibitor, also failed to reverse the reduction in cell viability in response to LCA treatment (Figure 3F). The phosphorylation of RIPK1, one of the key steps in the initiation of necroptosis,^46^ did not differ significantly under LCA treatment (Figure 3C–E). Furthermore, annexin V‐FITC and PI double staining showed that there was no significant difference between the treatment and control groups (Figure 3G). These data indicate that LCA significantly inhibited IEC‐6 cell proliferation without inducing apoptosis or necroptosis.

### 3.5 The Wnt/β‐catenin pathway was involved in the inhibition of cell proliferation

**FIGURE 4:**
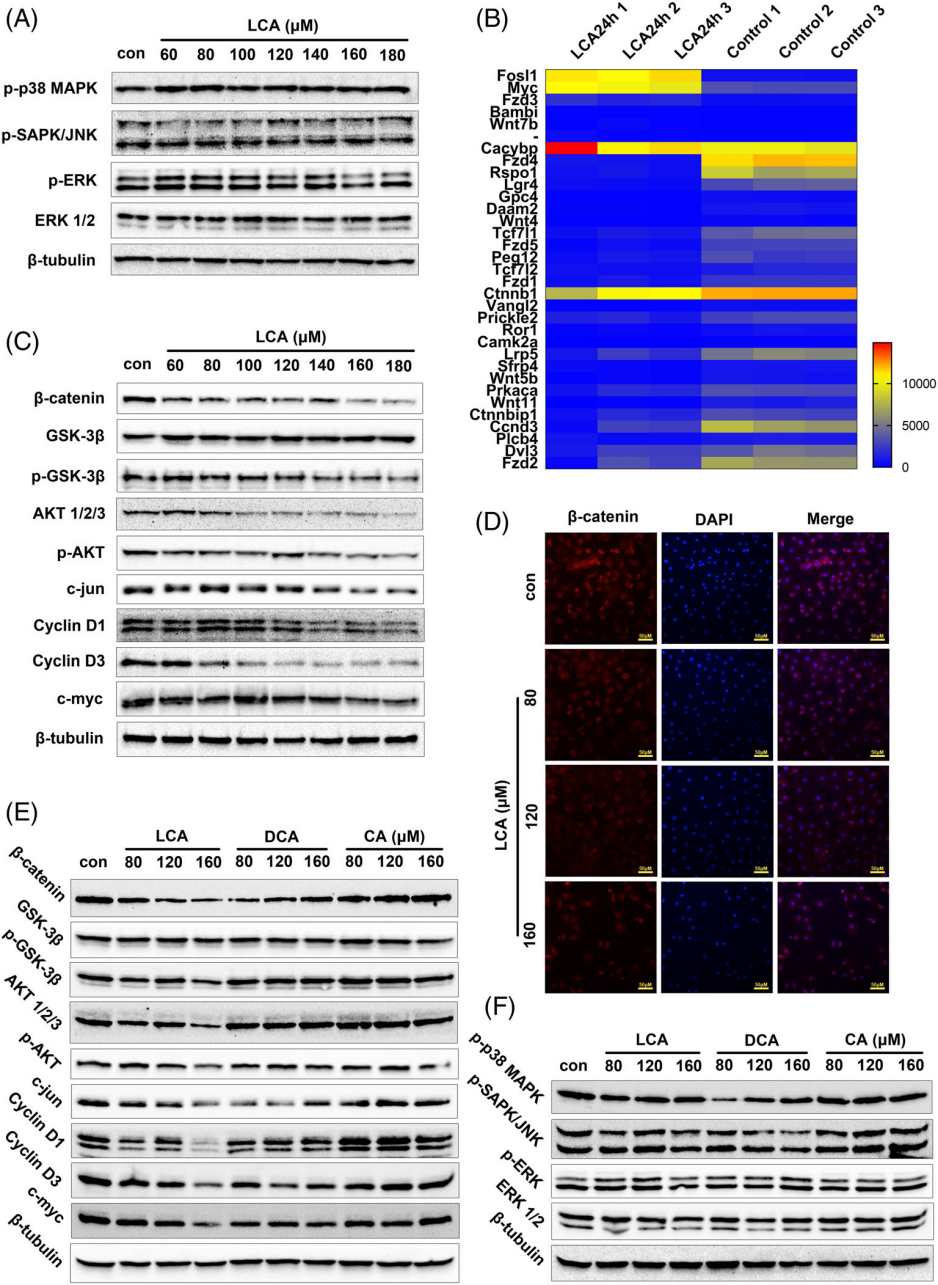
Wnt/β‐catenin pathway was involved in cell proliferation inhibition. (A) The expression or phosphorylation of key proteins in MAPK signalling pathway under treatment with lithocholic acid (LCA) (60–180 μM) treatment for 24 h. (B) Heat map of 33 DEGs related to Wnt/β‐catenin signalling pathway based on the limitation of log2|fold change| >1 and *p*adj ≤0.05. (C) The expression or phosphorylation of key proteins in Wnt/β‐catenin signalling pathway under treatment with LCA (60–180 μM) treatment for 24 h. (D) Immunofluorescence staining of β‐catenin (red) under treatment with LCA (80, 120 and 160 μM) for 24 h (×400). Cells were counterstained with DAPI (blue). (E,F) The expression or phosphorylation in Wnt/β‐catenin or MAPK signalling pathways under treatment with different does of LCA, DCA and CA for 24 h, respectively

Mechanistically, it has been reported that the MAPK and Wnt/β‐catenin pathways participate in cell proliferation via the G1/S phase.^47^, ^48^, ^49^ Under treatment with LCA (60–180 μM) for 24 h, the phosphorylation or expression of p‐SAPK/JNK (Thr183/Tyr185), p‐p38 MAPK (Thr180/Tyr182), p‐ERK (Thr202/Tyr204) and ERK 1/2, did not change significantly when compared with the control group (Figures 4A and S4A–D); similar results were acquired for both the 12 and 6 h treatments (Figure S5A,B), thus suggesting that the suppression of proliferation by LCA was independent of the MAPK pathways. Next, the activity of the Wnt/β‐catenin pathway was determined. As described earlier, the expression of cyclin D1 and cyclin D3 decreased in a dose‐dependent manner with LCA treatment (Figures 2D, 4C and S4L,M), which are the target genes for β‐catenin regulation and play a key role in cell cycle transition from G1 phase to S phase.^50^, ^51^, ^52^ A total of 33 DEGs related to the Wnt/β‐catenin signalling pathway were identified by RNA‐sequencing (Figure 4B). Under treatment with LCA (60–180 μM) for 24 h, the phosphorylation or expression of β‐catenin, p‐GSK‐3β (Ser 9), p‐AKT (Ser 473), AKT, c‐jun and c‐myc, were all reduced in a dose‐dependent manner (Figures 4C and S4E–K,N). These similar trends were observed after both 12 and 6 h of treatment (Figure S5C,D). Moreover, the immunofluorescence staining of β‐catenin also decreased in a dose‐dependent manner (Figure 4D). Wnt‐3A is the major cytokine responsible for β‐catenin activation.^53^, ^54^ IEC‐6 was treated with LCA (80, 120 and 160 μM), following by treatment with Wnt‐3A (20 ng/ml).^55^ The cell viability was tested using CCK‐8 assay. And Wnt‐3A can alleviate the inhibition of the cell proliferation at low concentrations of LCA, but not at high concentrations (Figure S6).

Next, we compared LCA with DCA or CA, and found that only LCA had an inhibitory effect on the Wnt/β‐catenin pathway (Figures 4E and S7A–J). DCA was more likely to act through the MAPK pathway while CA had no functional role in the Wnt/β‐catenin or MAPK pathway (Figure 4E,F, Figure S7A–N). Similar results were obtained after 12 and 6 h of treatment (Figure S5E–H). Collectively, our data suggest that LCA inhibited IEC‐6 cell proliferation in vitro by arresting cell cycle at the G1/S phase via the Wnt/β‐catenin pathway.

### 3.6 LCA triggered intestinal injury by inhibiting cell proliferation

**FIGURE 5:**
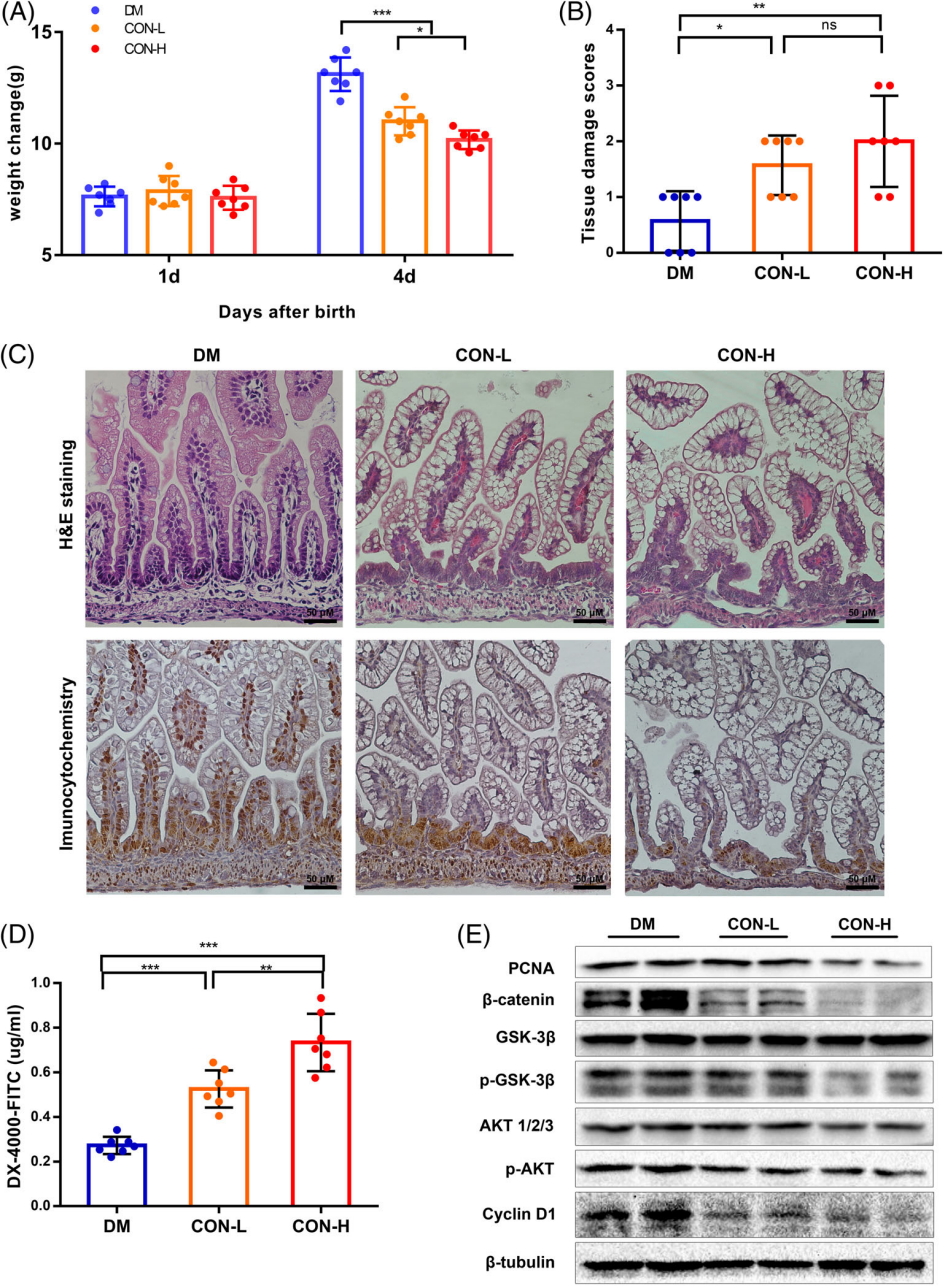
Lithocholic acid (LCA) triggers intestinal injury by inhibiting cell proliferation. (A) Changes in body weight of rats in DM, CON‐L and CON‐H groups. (B) Tissue damage scoring of rat ileum. (C) The H&E staining (up) and PCNA (brown)(down) immunohistochemistry staining of rat ileum (×400). (D) DX‐4000‐FITC levels in the serum of the DM, CON‐L and CON‐H groups. (E) Western blot analysis of PCNA and the key proteins in Wnt/β‐catenin signalling pathway in rat ileal tissue. *p <0.05; **p <0.01; ***p <0.001; ns, not significant. *p* values were obtained by one‐way ANOVA or Kruskal–Wallis test

The reduction of IEC cell proliferation leads to increased intestinal permeability and barrier dysfunction and is associated with a variety of intestinal diseases. Next, we investigated the effect of LCA on the rat intestine in vivo. Newborn rats were administered with low or high doses of LCA (namely the CON‐L or CON‐H groups) or fed with rat milk (namely DM group). Compared with the DM group, body weight gain was slower in the CON‐L and CON‐H groups (Figure 5A). Histological examination of the distal ileum showed more serious injury and higher damage scores in the CON‐L or CON‐H group than in the DM group (Figure 5B, C). In addition, the concentration of DX‐4000‐FITC in serum was significantly higher in the CON‐L and CON‐H group than in the DM group (Figure 5D). Compared with the DM group, the expression of PCNA and the number of PCNA‐positive epithelial cells all decreased markedly in the CON‐L and CON‐H group (Figure 5C,E, Figure S8A). Furthermore, the expression of β‐catenin, p‐GSK‐3β (Ser 9), p‐AKT (Ser 473), AKT was significantly decreased in the CON‐H and CON‐L groups compared with the DM group (Figure 5E, Figure S8B‐G). Similarly, the expression of cyclin D1, a target gene of β‐catenin, was also significantly decreased (Figure 5E, Figure S8H). Taken together, these data indicated that LCA inhibits intestinal cell proliferation and causes damage to the intestinal tissue by impairing Wnt/β‐catenin signalling.

### 3.7 Reduced cell proliferation due to Wnt/β‐catenin inhibition aggravated NEC

**FIGURE 6:**
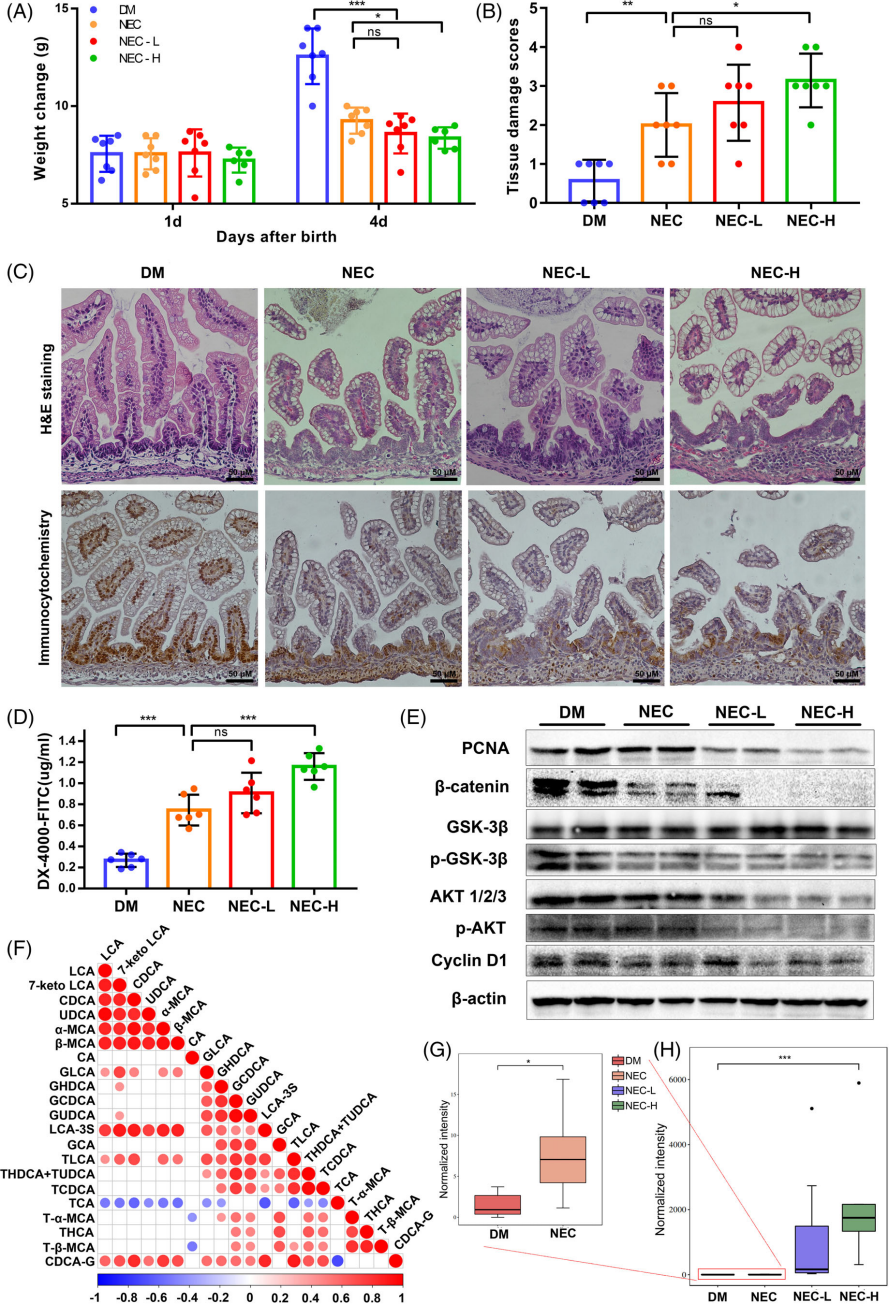
Decreased cell proliferation due to Wnt/β‐catenin inhibition aggravates necrotizing enterocolitis (NEC). (A) Changes in body weight of rats in DM, NEC, NEC‐L and NEC‐H groups. (B) Tissue damage scoring of rat ileum. (C) The H&E staining (Up) and PCNA (Brown)(Down) immunohistochemistry staining of rat ileum (×400). (D) DX‐4000‐FITC levels in the serum of DM, NEC, NEC‐L and NEC‐H groups. (E) Western blot analysis of PCNA and the key proteins in Wnt/β‐catenin signalling pathway in rat ileal tissue. (F) Correlation analysis among bile acids. Blank squares: *p* >0.05. Marked with red or blue (*p* <0.05) were the significant correlations. Positive correlations were shown in red; negative correlations were shown in blue. (G,H) Analysis of LCA levels in ileum tissues of DM, NEC, NEC‐L and NEC‐H groups. *p <0.05; **p <0.01; ***p <0.001; ns, not significant. *p* values were obtained by one‐way ANOVA or Kruskal–Wallis test

Finally, we investigated the effect of LCA on NEC. Neonatal rats were induced to developing NEC, treatment with high doses, low dose or without LCA, namely NEC‐H, NEC‐L or NEC group, respectively. DM group was fed by mother. The NEC‐H, NEC‐L and NEC groups exhibited slower weight gain and higher morbidity than the DM group (Figure 6A). Compared with the DM group, damage to the villi and submucosa of the distal ileum was evident in the NEC‐H, NEC‐L and NEC group (Figure 6C). The histology damage scores were highest in the NEC‐H group, followed by the NEC‐L, NEC and DM groups (Figure 6B). Similarly, the levels of DX‐4000‐FITC in serum followed the same trend (Figure 6D). Immunohistochemical staining showed that the proportion of PCNA‐positive enterocytes were significantly reduced in the NEC‐H and NEC‐L groups when compared to the DM or NEC groups (Figure 6C). Similarly, compared with the DM or NEC group, the expression of PCNA, β‐catenin, p‐GSK3β (Ser 9), p‐AKT (Ser 473), AKT and cyclin D1 decreased in the NEC‐L and NEC‐H groups (Figures 6E and S9A–H). Finally, the composition of BAs in intestinal tissues was tested by LC–MS/MS. The levels of LCA, 7‐keto LCA, CDCA, Urso‐DCA (UDCA), α‐Muricholic acid (α‐MCA), β‐MCA, glyco‐LCA(GLCA), LCA‐3‐sulfate sodium salt (LCA‐3S), Tauro‐LCA (TLCA) and CDCA‐G were increased in NEC, NEC‐L or NEC‐H group comparing with DM group (Figures 6F and S10A–I). As we expected, LCA was significantly higher in the NEC group (Figure 6G), and more obviously in the NEC‐L and NEC‐H group (Figure 6H). With AUC = 0.918, LCA was found to be a possible predictor of NEC in animal model by ROC curve (Figure S10J). Collectively, these data suggested that LCA plays a key role in the pathogenesis of NEC and acts by downregulating the Wnt/β‐catenin signalling, thus inhibiting the proliferation of enterocytes.

## 4 DISCUSSION

**FIGURE 7:**
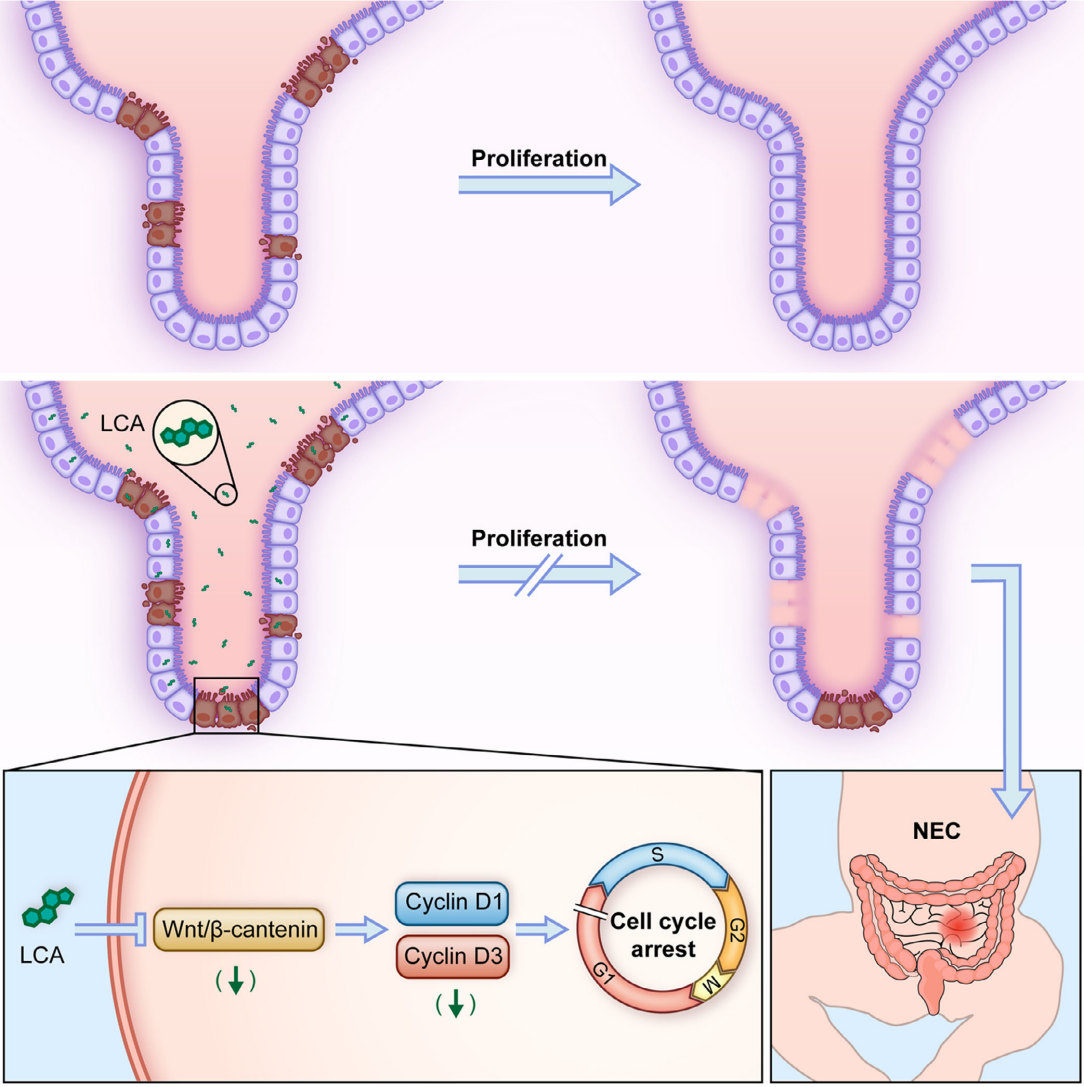
Model indicated that LCA exacerbates necrotizing enterocolitis (NEC) by inhibiting enterocyte proliferation through arresting at G1/S phase in a β‐catenin‐dependent manner

In this study, we confirmed that LCA aggravated NEC by inhibiting the proliferation of enterocytes through arresting at G1/S phase in a β‐catenin‐dependent manner (Figure 7). To our knowledge, our study is the first to illustrate the effect of LCA on Wnt/β‐catenin signalling in NEC. We also found that LCA had a more obvious inhibitory role in enterocyte proliferation than DCA or CA.

BAs are produced by the liver, secreted into the duodenum and then undergo the conversion of primary BAs (CDCA, CA) to secondary BAs (DCA, LCA) by the gut microbiota; they are then recycled back to the liver in the terminal ileum via the enterohepatic circulation.^56^, ^57^, ^58^ When NEC occurs, the distal ileum is damaged; this can lead to the obstruction of the enterohepatic circulation, thus causing difficulties in the recycling of BAs and the consequent accumulation of BAs.^25^, ^59^ In turn, the accumulation of BAs can aggravate intestinal damage.^28^, ^60^ The chemical structure and properties of BAs are diverse; therefore, the role of BAs differs across many diseases. It has been reported that CDCA can stimulate the release of inflammatory factors and lead to increased intestinal permeability^61^ while UDCA has been shown to inhibit apoptosis and alleviate NEC‐induced injury.^62^ Other research has shown that tauroursodeoxycholic acid alleviates intestinal injury by inhibiting endoplasmic reticulum stress in NEC.^63^ In our experiments, we found that LCA, but not DCA and CA, significantly inhibited cell proliferation by inhibiting Wnt/β‐catenin signalling and that LCA supplementation led to the exacerbation of NEC. Our research provided a new perspective for the pathogenesis of NEC. Unlike our results, some previous studies have indicated that LCA can result in carcinogenesis.^64^, ^65^ In contrast, Sato et al. reported that the increased lifespan in humans correlates somewhat with increased levels of LCA in the intestine.^66^ Moreover, Huang et al. reported that LCA can attenuate inflammation in the early phase of experimental NEC by regulating the pregnane X receptor/toll‐like receptor 4 signalling pathway, but ultimately failed to alleviate histologic severity.^67^ General speaking, LCA may play a dual role in the progression of NEC progression via different signalling pathways; however, eventually the harm outweighs the benefits. Further studies are now required to further elucidate this possibility.

The intestinal epithelium undergoes renewal every 3–5 days in humans and every 2–3 days in mice.^68^, ^69^ When the epithelial layer is damaged, epithelial regeneration and cell proliferation occur rapidly to repair the defective cell barrier.^70^ Thus, the inhibition of proliferation can result in disruption of this renewal process, thus leading to the initiation or exacerbation of disease. Notably, most inflammatory bowel diseases, including NEC, Crohn's disease and ulcerative colitis, are closely associated with impaired enterocyte proliferation and disruption of the intestinal barrier.^71^, ^72^, ^73^ Our findings confirmed the possibility that LCA resulted in severe disruption of the intestinal barrier due to inhibition of intestinal epithelial renewal and regeneration.

The Wnt/β‐catenin pathway is involved in a wide range of physiological and pathological mechanisms.^74^, ^75^, ^76^ It is well known that the Wnt/β‐catenin signalling pathway plays a key role in embryonic intestinal development, homeostasis of the adult intestine and pathogenesis of intestinal cells.^77^, ^78^ Signalling molecules associated with the Wnt/β‐catenin pathway are expressed along the villi axis and regulate epithelial homeostasis between cell proliferation and differentiation in both a spatial and temporal manner.^79^ Previous studies have shown that Wnt/β‐catenin‐dependent mechanisms stimulate epithelial cell proliferation.^80^, ^81^ Therefore, inhibition of the Wnt/β‐catenin pathway leads to the blockade of enterocyte proliferation, and subsequently, the aggravation of NEC.^17^, ^21^, ^82^, ^83^ Our data are consistent with these previous findings. However, we also discovered that LCA, a natural metabolite of the intestinal flora, can specifically regulate the Wnt/β‐catenin pathway. However, the detailed underlying mechanisms responsible for how LCA inhibits the Wnt/β‐catenin pathway have yet to be elucidated.

Some limitations exist in the current study that need to be considered. On the one hand, LCA is an important metabolite of bacteria in the gut; a large number of studies have shown that the onset of NEC is closely related to an imbalance in the intestinal microecology.^11^, ^84^ The microbiome has an important influence on the pathogenesis of NEC. Intestinal stem cells have a very close relationship with intestinal cell proliferation and the Wnt/β‐catenin pathway.^85^, ^86^ However, the specific roles of the intestinal flora and intestinal stem cells were not explored in this study. Further experiments now need to address these limitations. In summary, the present study revealed an important role for LCA in the pathogenesis of NEC. LCA caused a deterioration of NEC by inhibiting intestinal cell proliferation through the inhibition of the Wnt/β‐catenin pathway. Accordingly, this study indicates a possible role for LCA as a predictors of NEC.

## CONFLICT OF INTEREST

The authors declare that they have no conflict of interest.

## AUTHOR CONTRIBUTIONS

Zhoushan Feng, Chunhong Jia, Xiaojun Lin, Yaoyong Chen and Fan Wu performed study conception; Zhoushan Feng, Chunhong Jia, Xiaojun Lin, Hu Hao and Sitao Li were responsible for experimental design and implementation; Zhoushan Feng, Chunhong Jia, Xiaojun Lin, Yaoyong Chen and Fei Li provided acquisition, analysis and interpretation of data and drafted the article; Xin Xiao, Yaoyong Chen and Fan Wu revised the article and provided material support. All authors read and approved the final paper.

## Supporting information


**Appendix** S1: Supporting Information

## Data Availability

Data for this study are available from the corresponding author on reasonable request.
