# Supplementary material for: The inhibition of enterocyte proliferation by lithocholic acid exacerbates necrotizing enterocolitis through downregulating the Wnt/β‐catenin signalling pathway
Source: Cell Prolif. 2022 Apr 20;55(5):e13228. doi: 10.1111/cpr.13228 (PMC9136529; doi:10.1111/cpr.13228)
Supplement: Supplementary file 1 — Appendix S1: Supporting Information [file CPR-55-e13228-s001.docx]

**Supplementary Information**

**The inhibition of enterocyte proliferation by lithocholic acid exacerbates necrotizing enterocolitis by downregulating the Wnt/β-catenin signaling pathway**

Zhoushan Feng^1, 2*^, Chunhong Jia^2, 3*^, Xiaojun Lin^2*^, Hu Hao^1^, Sitao Li^1^, Fei Li^1^, Qiliang Cui^2^, Yaoyong Chen^2^, Fan Wu^2, 3^ and Xin Xiao^1^

1. Department of Pediatrics, The Sixth Affiliated Hospital, Sun Yat-sen University, Guangzhou, 510655, China.
2. Department of Pediatrics, The Third Affiliated Hospital of Guangzhou Medical University, Guangzhou, 510140, China.
3. Key Laboratory for Major Obstetric Diseases of Guangdong Province, Guangzhou, 510140, China.

*These authors contributed equally to this study

**Table S1**

**Clinical characteristics of NEC patients at stage II or III**

| Characteristic | Stage II (n = 9) | Stage III (n = 7) | p value |
| --- | --- | --- | --- |
| GA (weeks) | 31.05 ± 3.28 | 31.53 ± 3.78 | 0.803 |
| BW (g) | 1534.44 ± 656.29 | 1432.86 ± 432.36 | 0.745 |
| Female (%) | 2/9 (22.22%) | 4/7 (57.14%) | 0.302 |
| Apgar score at 5 min | 10 (10, 10) | 10 (9, 10) | 0.596 |
| Enteral feeding within 24 h (%) | 5/9 (55.56%) | 5/7 (71.43%) | 0.633 |
| Type of feeding: |  |  |  |
| MM | 0 | 0 | 0.633 |
| PF | 4 | 2 |  |
| MM+PF | 5 | 5 |  |
| Total BAs within 24 h after birth (umol/L) | 9.81 (6.66, 12.50) | 4.89 (3.64, 5.82) | < 0.001 |
| Total BAs at NEC diagnosis (umol/L) | 14.22 (12.9, 18.4) | 10 (8.56, 16.93) | 0.299 |
| Total bilirubin (umol/L) | 107.76 ± 50.32 | 99.51 ± 22.71 | 0.712 |
| Direct bilirubin (umol/L) | 11.96 ± 6.84 | 11.50 ± 3.55 | 0.882 |
| Time at blood test (d) | 9 (6, 12) | 11(10, 20) | 0.218 |
| RBC transfusion (%) | 4/9 (44.44%) | 4/7 (57.14) | 1.000 |
| NASIDs (%) | 1/9 (11.11%) | 1/7 (14.29%) | 1.000 |

Data are expressed as medians (25%, 75%), mean ± SD or numbers (%). NEC, necrotizing enterocolitis; GA, gestational age; BW, birth weight; BAs, bile acids; MM, mother`s milk; PF, preterm formula; RBC, red blood cell; NASIDs, nonsteroidal anti-inflammatory drugs. P values were calculated using Student`s t-test, Wilcoxon test, chi square or Fisher's exact.

**Table S2**

**Clinical characteristics of NEC patients requiring surgical or non-surgical treatment**

| Characteristic | Non-surgery(n = 9) | Surgery (n = 7) | p value |
| --- | --- | --- | --- |
| GA (weeks) | 31.67 ± 3.59 | 30.73 ± 3.35 | 0.626 |
| BW (g) | 1631.11 ± 641.7 | 1308.57 ± 398.15 | 0.294 |
| Female (%) | 6/9 (66.67%) | 4/7 (57.14%) | 1.000 |
| Apgar score at 5 min | 10 (10, 10) | 10 (9, 10) | 0.596 |
| Enteral feeding within 24 h (%) | 6/9 (66.67%) | 4/7 (57.14%) | 1.000 |
| Type of feeding: |  |  |  |
| MM | 0 | 0 | 0.302 |
| PF | 2 | 4 |  |
| MM+PF | 7 | 3 |  |
| Total BAs within 24 h after birth (umol/L) | 5.35 (4.43, 5.97) | 9.7 (6.28, 11.09) | 0.02 |
| Total BAs at NEC diagnosis (umol/L) | 13.38 (11.90, 17.39) | 11.60 (9.99, 22.25) | 0.958 |
| Total bilirubin (umol/L) | 104.61 ± 50.88 | 103.56 ± 21.91 | 0.962 |
| Direct bilirubin (umol/L) | 11.69 ± 6.86 | 11.84 ± 3.51 | 0.960 |
| Time at blood test (d) | 10 (8, 12) | 11 (8.5, 20) | 0.518 |
| RBC transfusion (%) | 3/9 (33.33%) | 5/7 (71.43%) | 0.315 |
| NASIDs (%) | 0 | 2/7 (28.57%%) | - |

Data are expressed as medians (25%, 75%), mean ± SD or numbers (%). NEC, necrotizing enterocolitis; GA, gestational age; BW, birth weight; BAs, bile acids; MM, mother`s milk; PF, preterm formula; RBC, red blood cell; NASIDs, nonsteroidal anti-inflammatory drugs. P values were calculated using Student`s t-test, Wilcoxon test, chi square or Fisher's exact.


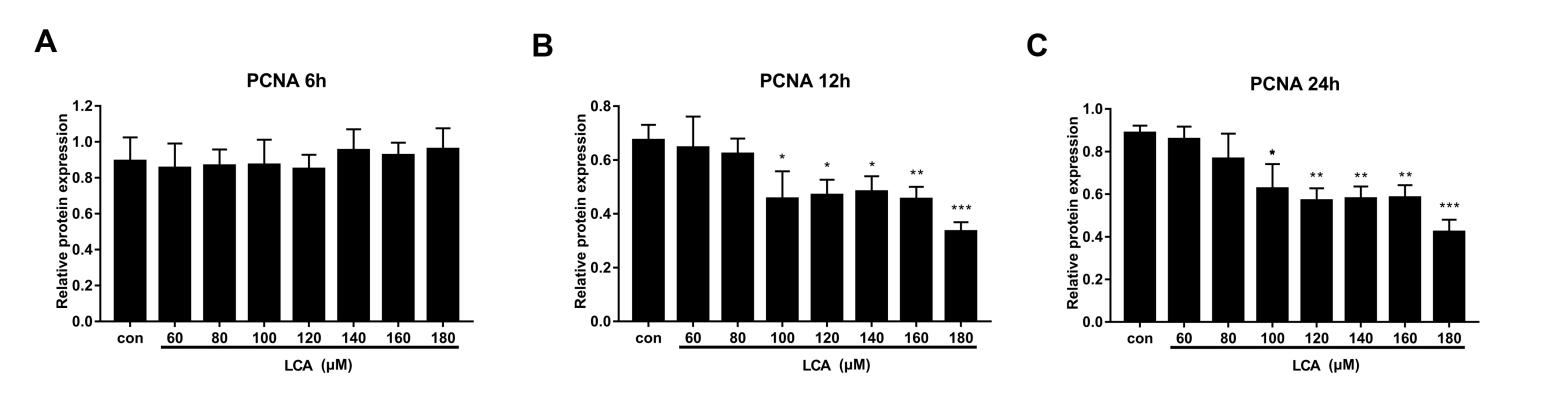


**Figure S1. (A-C)** Quantification of PCNA relative protein expression at 6, 12 and 24 hours. * p < 0.05, ** p < 0.01, *** p < 0.001 compared to control by one-way ANOVA; Data provided are the mean ± SD from at least three independent experiments, and bar graphs represent the mean with error bars indicating SD.


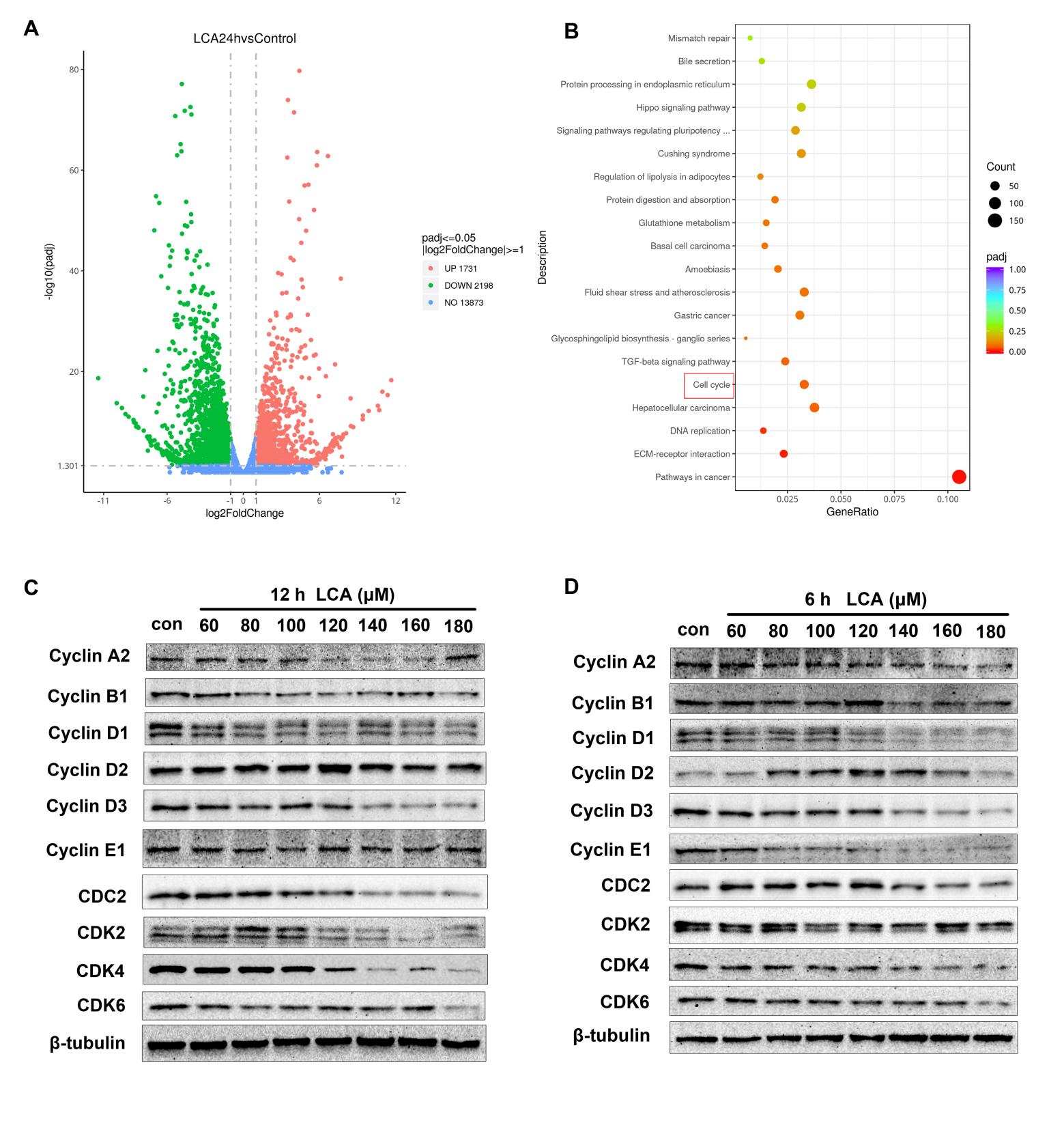


**Figure S2. (A)** Volcano plot of differentially expressed genes (DEGs) based on the limitation of log2 |fold change| > 1 and adjust p-value (padj) ≤ 0.05. **(B)** Scatter plot of top 20 KEGG pathways enrichment of DEGs. **(C, D)** Expression of cyclins and cyclin-dependent kinases under treatment with different dose of LCA (60 μM-180 μM) for 12 h and 6 h.


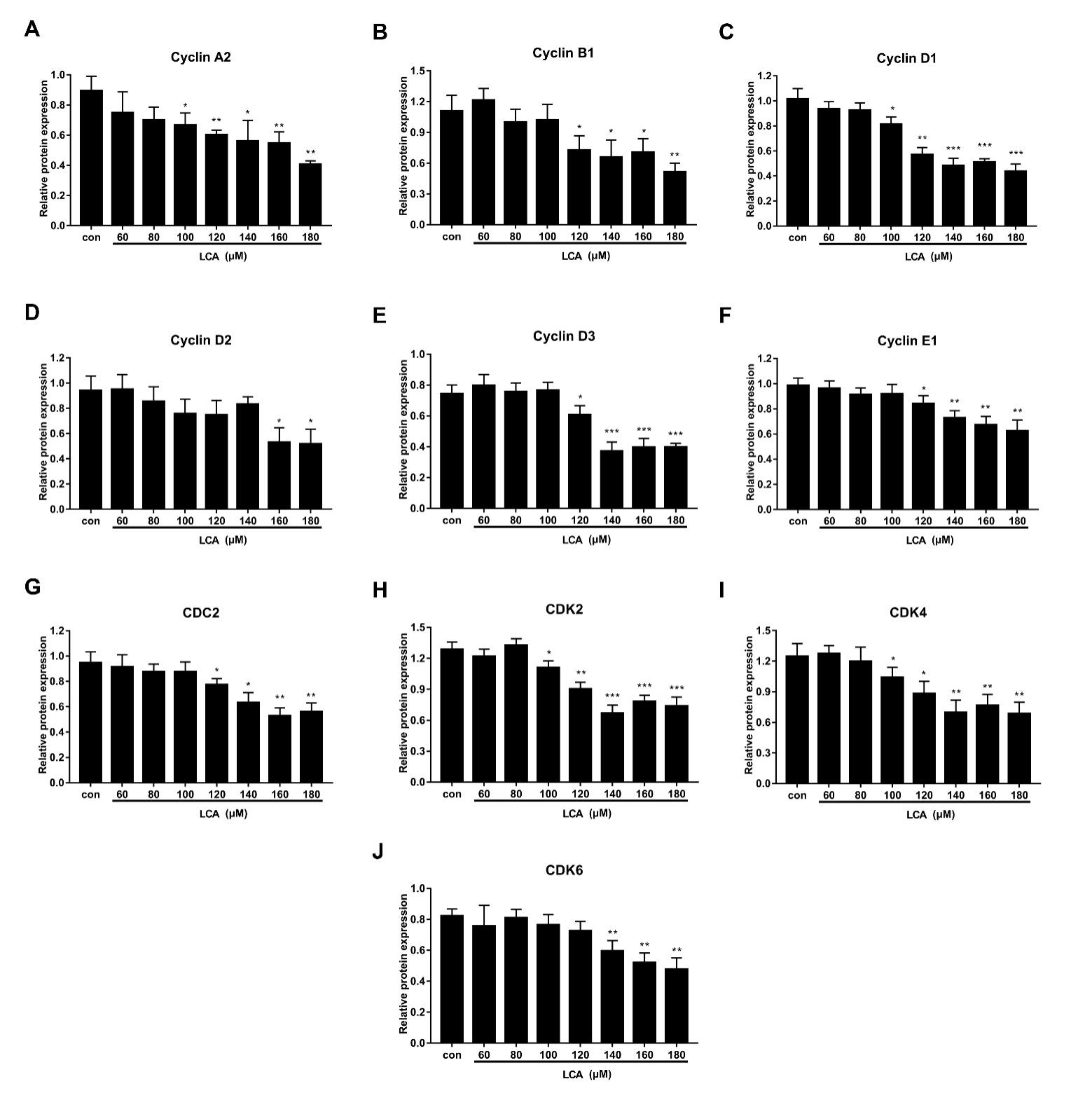


**Figure S3. (A-J)** Quantification of cyclin A2, cyclin B1, cyclin D1, cyclin D2, cyclin D3, cyclin E1, CDC2, CDK4 and CDK6 relative protein expression under treatment with different dose of LCA (60 μM-180 μM) for 24 h. * p < 0.05, ** p < 0.01, *** p < 0.001 compared to control by one-way ANOVA; Data provided are the mean ± SD from at least three independent experiments, and bar graphs represent the mean with error bars indicating SD.


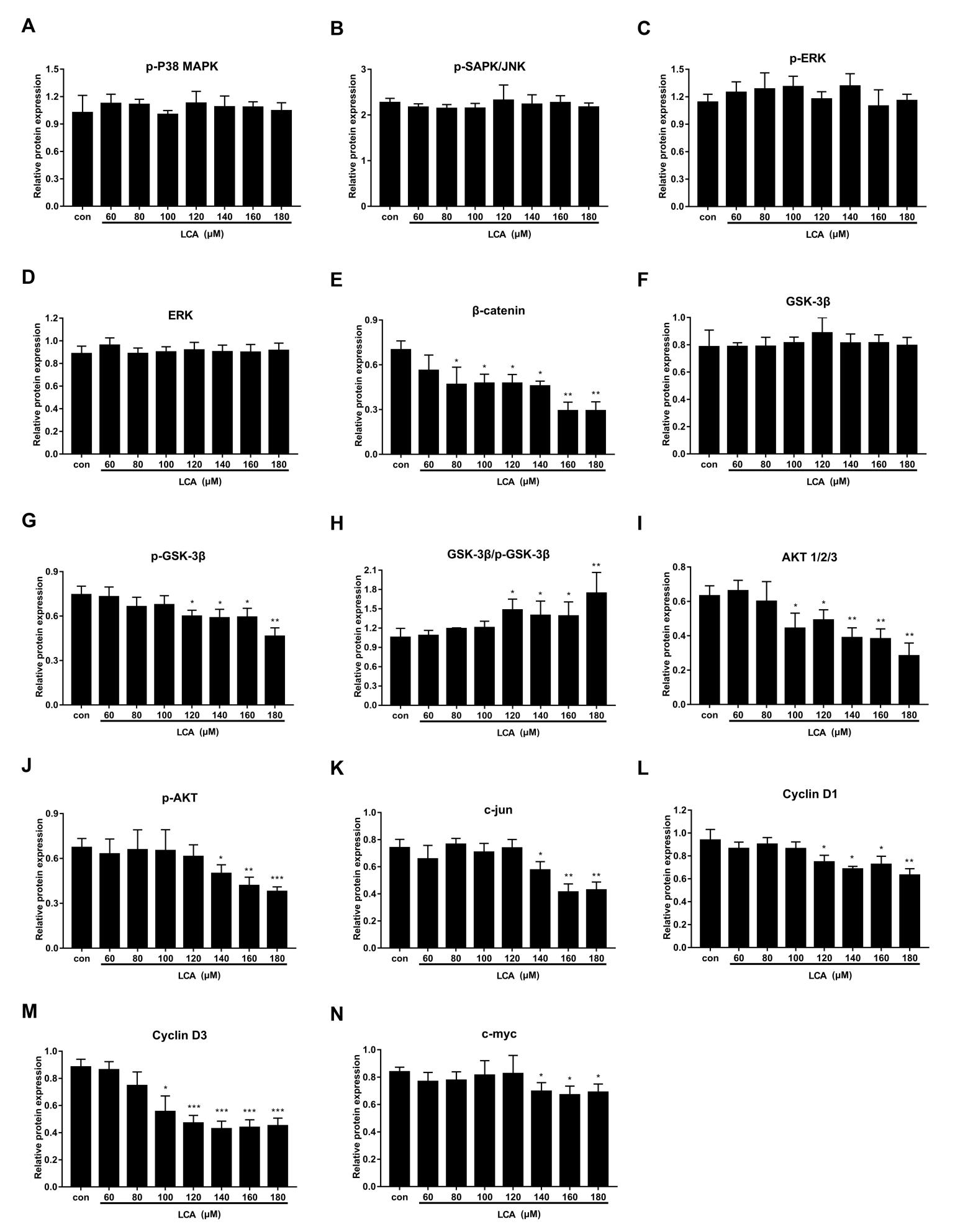


**Figure S4. (A-D)** Quantification of p-P38 MAPK, p-SAPK/JNK, p-ERK, ERK relative protein expression under treatment with different dose of LCA (60 μM-180 μM) for 24 h. **(E-N)** Quantifying the expression of relative proteins such as β-catenin, GSK3β, p-GSK3β, GSK3β/p-GSK-3β, p-AKT, AKT, c-jun, cyclin D1, cyclin D3, and c-myc. * p < 0.05, ** p < 0.01, *** p < 0.001 compared to control by one-way ANOVA; Data provided are the mean ± SD from at least three independent experiments, and bar graphs represent the mean with error bars indicating SD.


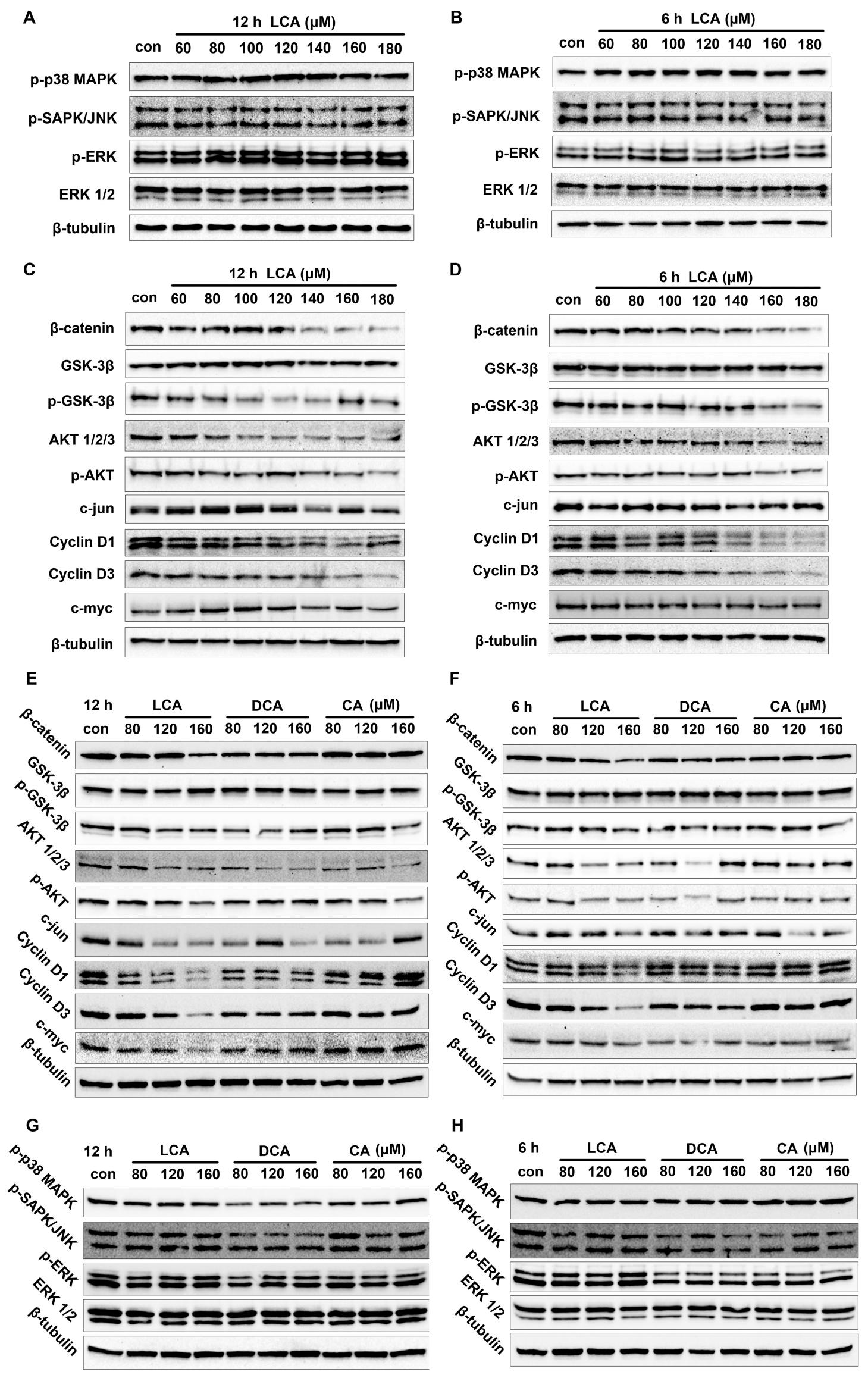


**Figure S5.** The expression or phosphorylation of key proteins in MAPK signaling pathway **(A, B)** and Wnt/β-catenin signaling pathway **(C, D)** under treatment with LCA (60 μM-180 μM) treatment for 12 h or 6 h. The expression or phosphorylation of key proteins in Wnt/β-catenin signaling pathway **(E, F)** and MAPK signaling pathway **(G, H)** under treatment with different does (80 μM, 120 μM and 160 μM) of LCA, DCA and CA for 12 h or 6 h, respectively.


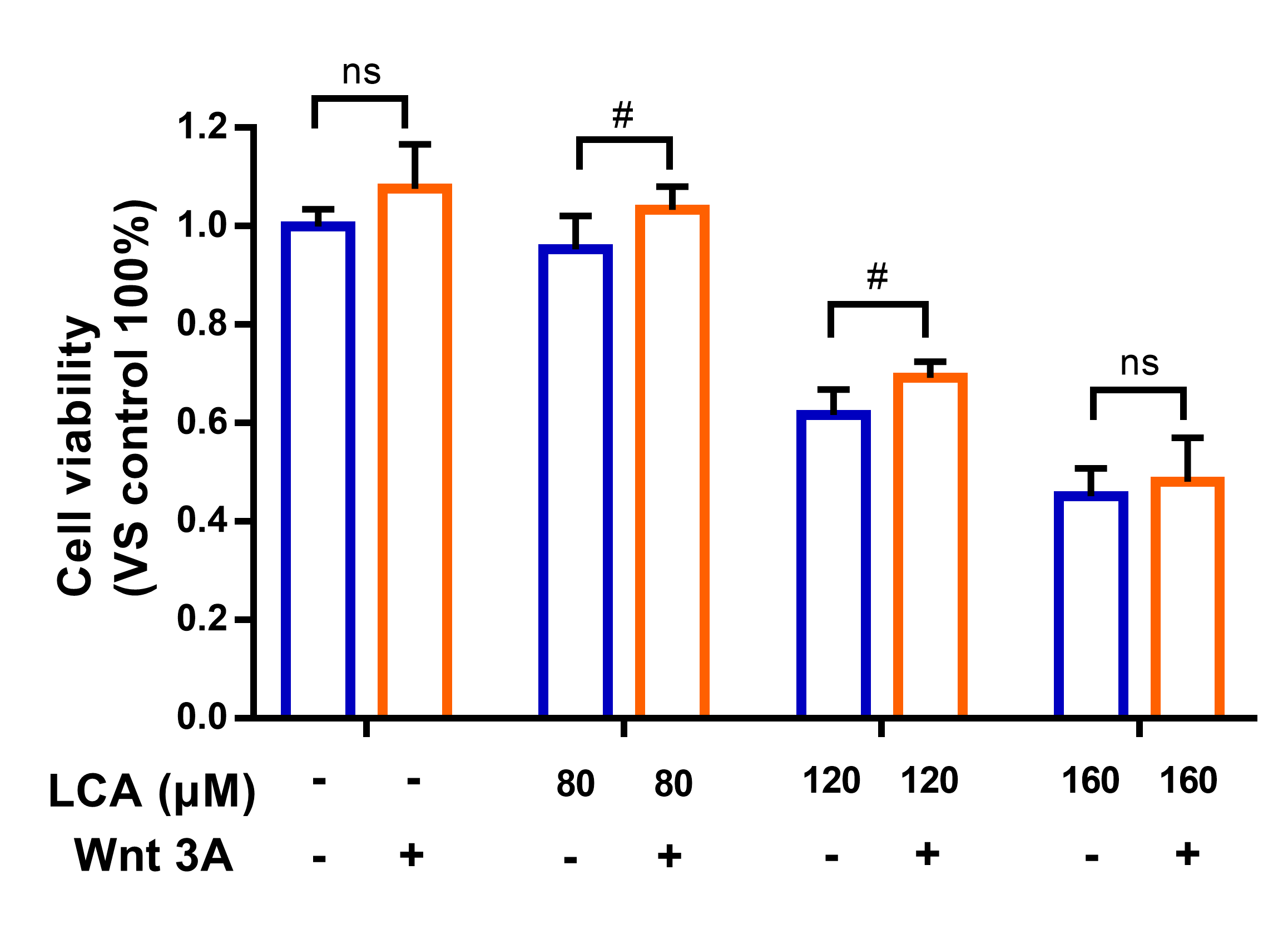


**Figure S6.** IEC-6 cell proliferation was tested by CCK-8 assay under pretreatment with LCA (80 μM, 120μM and 160 μM), following by treatment with Wnt-3A (20 ng/ml). ^#^ p < 0.05 compared to LCA by Student's t-test; ns: not significant. Data provided are the mean ± SD from at least three independent experiments, and bar graphs represent the mean with error bars indicating SD.


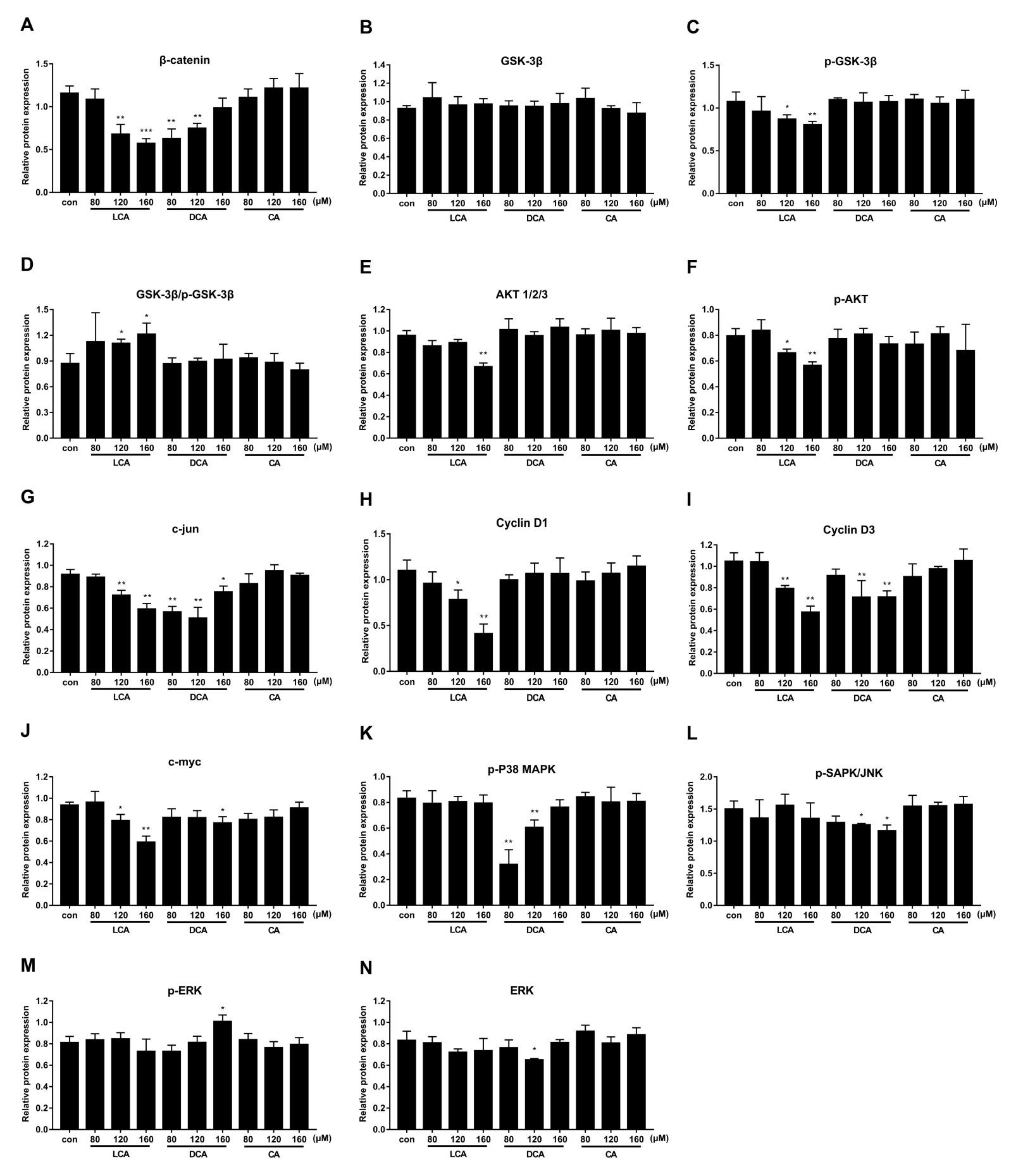


**Figure S7. (A-J)** Quantification of the expression or phosphorylation in Wnt/β-catenin signaling pathways under treatment with different does of LCA, DCA and CA for 24 h, respectively. **(K-N)** Quantifying the expression or phosphorylation in MAPK signaling pathways. * p < 0.05, ** p < 0.01, *** p < 0.001 compared to control by one-way ANOVA; Data provided are the mean ± SD from at least three independent experiments, and bar graphs represent the mean with error bars indicating SD.


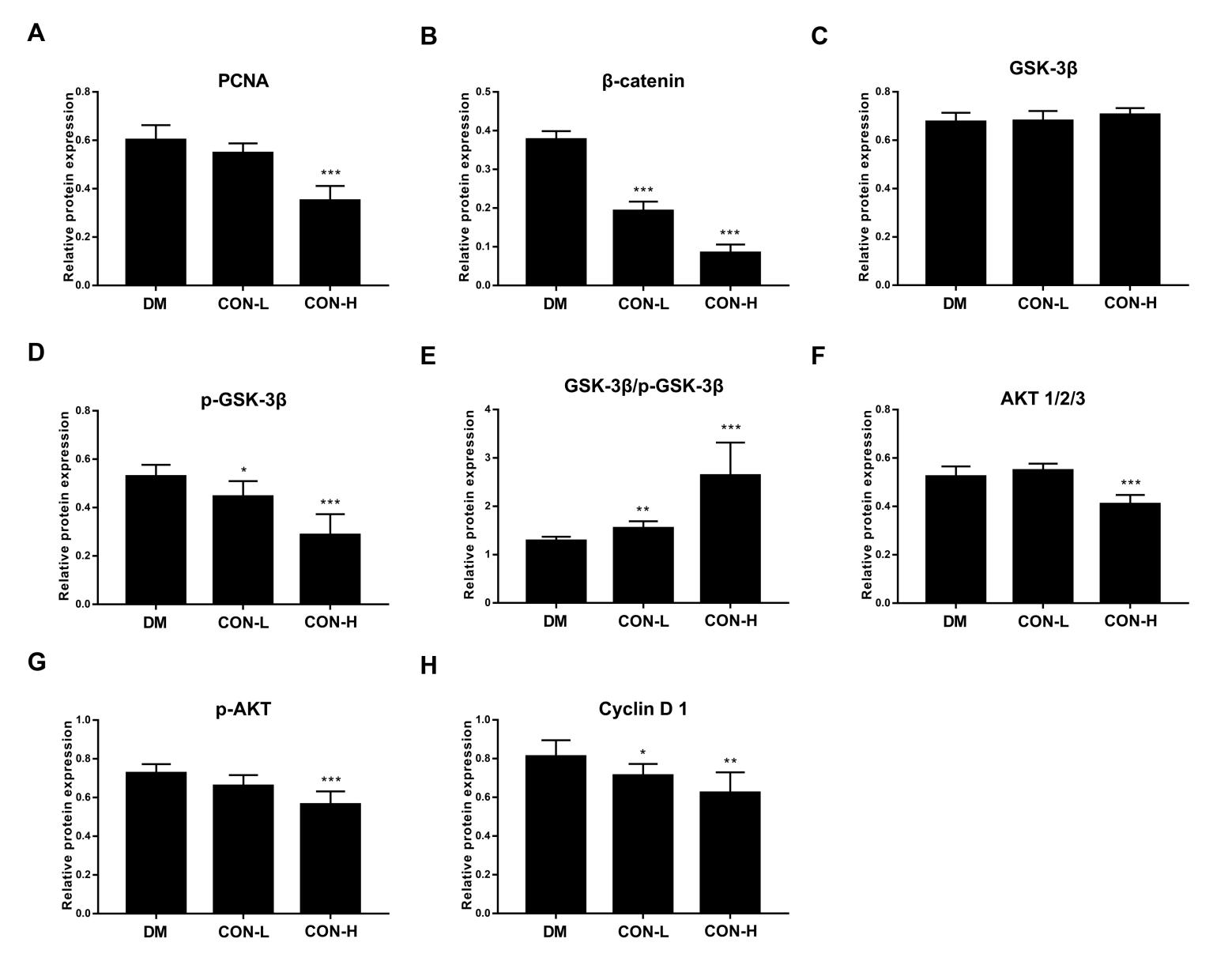


**Figure S8. (A)** Quantification of PCNA relative protein expression in rat ileal tissue. **(B-H)** Quantifying the expression of relative proteins such as β-catenin, GSK3β, p-GSK3β, GSK3β/p-GSK-3β, p-AKT, AKT and cyclin D1. * p < 0.05, ** p < 0.01, *** p < 0.001 compared to DM group by one-way ANOVA; Data provided are the mean ± SD from at least three independent experiments, and bar graphs represent the mean with error bars indicating SD.


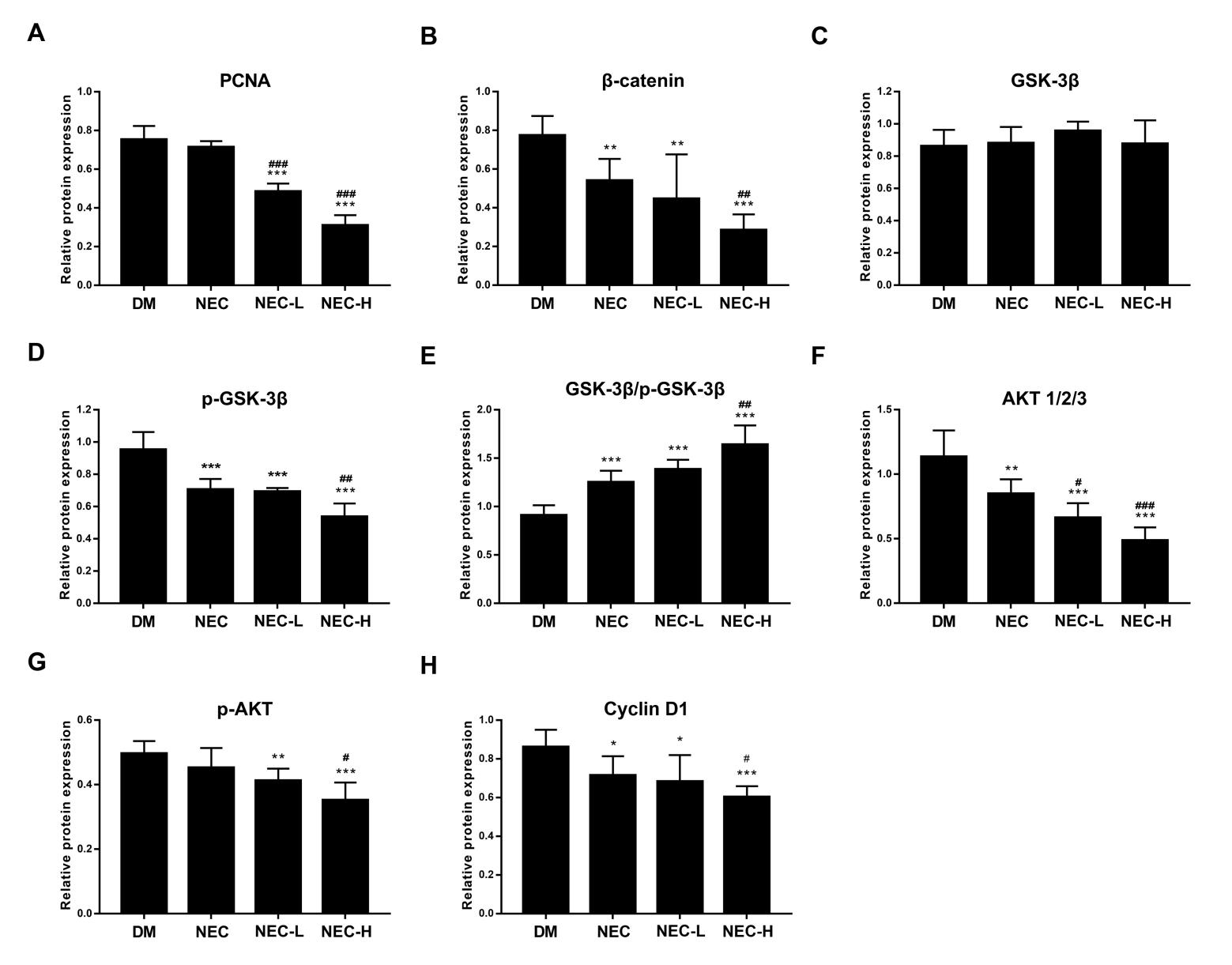


**Figure S9. (A)** Quantification of PCNA relative protein expression in DM, NEC, NEC-L and NEC-H group. **(B-H)** Quantification of the expression or phosphorylation of Wnt/β-catenin signaling pathways in rat ileal tissue. * p < 0.05, ** p < 0.01, *** p < 0.001 compared to DM group by one-way ANOVA, while ^#^ p < 0.05, ^##^ p < 0.01, ^###^ p < 0.001 compared to NEC group by one-way ANOVA; Data provided are the mean ± SD from at least three independent experiments, and bar graphs represent the mean with error bars indicating SD.


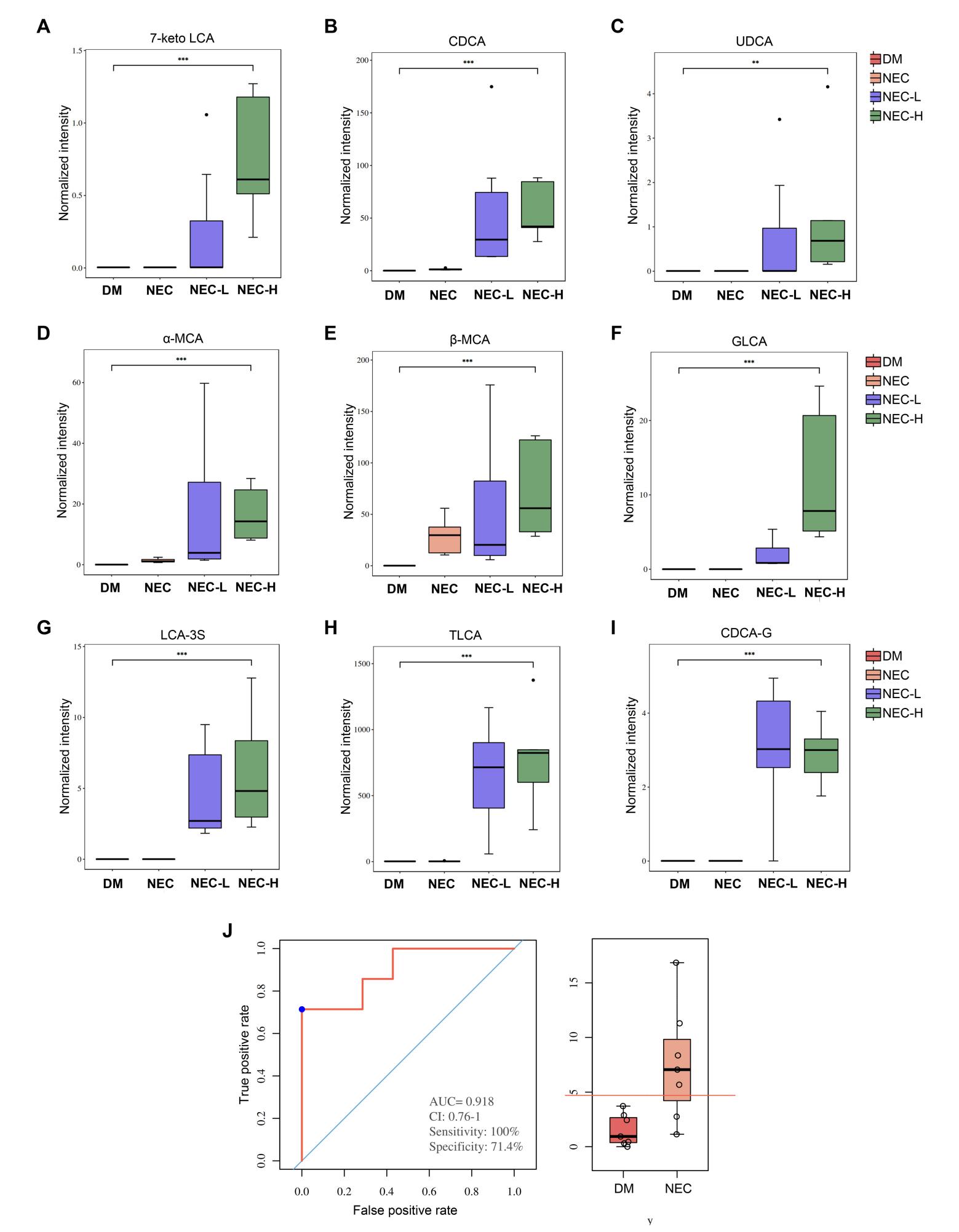


**Figure S10. (A-I)** The levels of 7-keto LCA, CDCA, Urso-DCA (UDCA), α-Muricholic acid (α-MCA), β-MCA, Glyco-LCA(GLCA), LCA-3-sulfate sodium salt (LCA-3S), Tauro-LCA (TLCA) and CDCA-G in ileum tissues of DM, NEC, NEC-L and NEC-H groups. **(J)** Diagnostic value of LCA for NEC according to ROC curves. ***P < 0.001. P values were calculated using Kruskal-Wallis test.
